# Triazolyl Conjugated (Oligo)Phenothiazines Building Blocks for Hybrid Materials—Synthesis and Electronic Properties

**DOI:** 10.3390/molecules26102950

**Published:** 2021-05-15

**Authors:** Hilla Khelwati, Adam W. Franz, Zhou Zhou, Werner R. Thiel, Thomas J. J. Müller

**Affiliations:** 1 Institut für Organische Chemie und Makromolekulare Chemie, Heinrich-Heine-Universität Düsseldorf, Universitätsstrasse 1, D-40225 Düsseldorf, Germany; Hilla.Khelwati@uni-duesseldorf.de (H.K.); adam.franz@hhu.de (A.W.F.); 2 Fachbereich Chemie, Technische Universität Kaiserslautern, Erwin-Schrödinger-Str. 54, D-67663 Kaiserslautern, Germany; zhou.zhou@gmx.de

**Keywords:** absorption, Cu-catalyzed alkyne-azide cycloaddition, fluorescence, mesoporous hybrid materials, phenothiazine, triazole

## Abstract

The Cu-catalyzed alkyne-azide 1,3-dipolar cycloaddition variant provides a highly efficient entry to conjugated triazolyl-substituted (oligo)phenothiazine organosilicon derivatives with luminescence and reversible redox characteristics. Furthermore, by in-situ co-condensation synthesis several representative mesoporous MCM-41 type silica hybrid materials with embedded (oligo)phenothiazines are prepared and characterized with respect to their structural and electronic properties. The hybrid materials also can be oxidized to covalently bound embedded radical cations, which are identified by their UV/Vis absorption signature and EPR signals.

## 1. Introduction

The mechanistic details of 1,3-dipolar cycloaddition were elucidated by Huisgen and his group almost six decades ago [1]. Ever since, it has become a versatile reaction for the formation of five-membered heterocycles, even in complex natural pro-duct syntheses [2]. However, it took almost 40 years for 1,3-dipolar cycloadditions, among other highly atom economical (cyclo)additions, to lead to the formulation of the “click chemistry” concept, coined by Sharpless and coauthors [3]. Although the synthesis of 1,2,3-triazoles by 1,3-dipolar cycloaddition of organic azides to alkynes was established and mechanistically well described [4], the regioselectivity of the pericyclic process is often poor or even absent for terminal alkynes and organic azides. Changing the mechanism from the thermal process to a stepwise organometallic copper-catalyzed process, named Meldal-Sharpless reaction [5,6] or copper-catalyzed alkyne-azide cycloaddition (CuAAC) [7], gave rise to the highly selective formation of 1,4-disubstituted triazoles. Mechanistic elucidation [8], catalyst design [9], and implementation into multicomponent methodologies [10] mark basic expansions of CuAAC over the years.

Besides application in drug discovery [11] and lead discovery [12] in medicinal chemistry [13] and bioorganic chemistry [14,15] due to the operational simplicity and broad tolerance for functional groups, CuAAC found rapid use as a valuable ligation tool in polymer and materials sciences, either for polymer analog transformation or for polymerization, as emphasized in several reviews since the early days [16,17,18,19,20,21,22,23]. Furthermore, meso-porous silica hybrid materials have also been successfully functionalized by azides employing CuAAC as a postsynthetic grafting tool [24] for internal and external surface deco-ration in biosensing applications [25]. Recently, we used CuAAC for preparing triethoxysilylated substituted fluorophore-containing precursors for the synthesis of Nile Red-embedded mesoporous silica hybrids as solvent polarity sensors that also operate in aqueous media [26]. Furthermore, we expanded the CuAAC approach for the synthesis of RGB fluorophore precursors that were used for mesoporous silica hybrid materials for energy down-converting phosphors and application in monolith coated light emitting diodes [27]. Based upon our experience in covalently anchoring phenothiazine electrophores in silica hybrid materials [28], either by postsynthetic grafting with carbamate linkers [29] or in-situ synthesis of the hybrid materials using the more hydrolysis stable urea linkers [30], we reasoned that CuAAC of phenothiazinyl alkynes with azido functiona-lized triethoxysilanes would provide highly luminescent, reversible redox systems that can be employed in hybrid silica in-situ synthesis. Herein we report the synthesis by CuAAC and electronic properties of triazolyl conjugated oligophenothiazines as luminescent electrophore precursors.

## 2. Results and Discussion

### 2.1. Synthesis

**Scheme 1:**
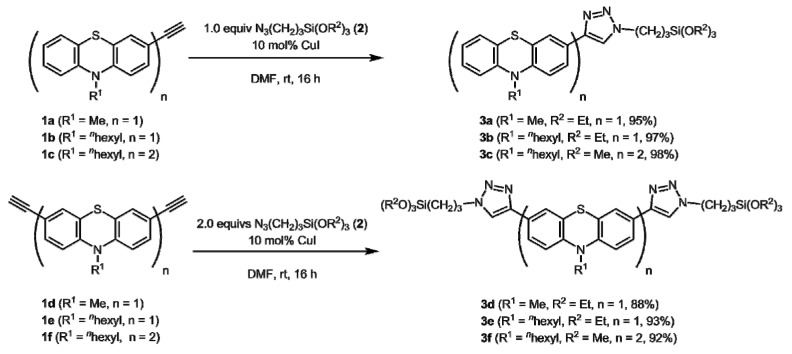
CuAAC synthesis of linear triazolyl conjugated triethoxylsilylpropyltriazolyl (oligo)phenothiazines **3**.

Our synthesis of linear conjugated triazolylsubstituted (oligo)phenothiazine precursors for hybrid materials’ synthesis bearing triethoxylsilyl propyl groups commences from ethynylated (oligo)phenothiazines **1** [31,32,33,34]. Mono- and diethynylated (oligo)phenothiazines **1** undergo the CuAAC at room temp in DMF with (3-azidopropyl)tri-alkoxysilanes **2 [35]** to give conjugated triazolylsubstituted (oligo)phenothiazines **3** in excellent yield as oils (Scheme 1). Both triethoxy- and trimethoxysilyl derivatives **3** are readily obtained as well as methyl- and *n*-hexyl-substituted phenothiazine derivatives. The structures of compounds **3** were unambiguously assigned by IR, ^1^H- and ^13^C-NMR spectroscopy and mass spectrometry.

**Scheme 2:**
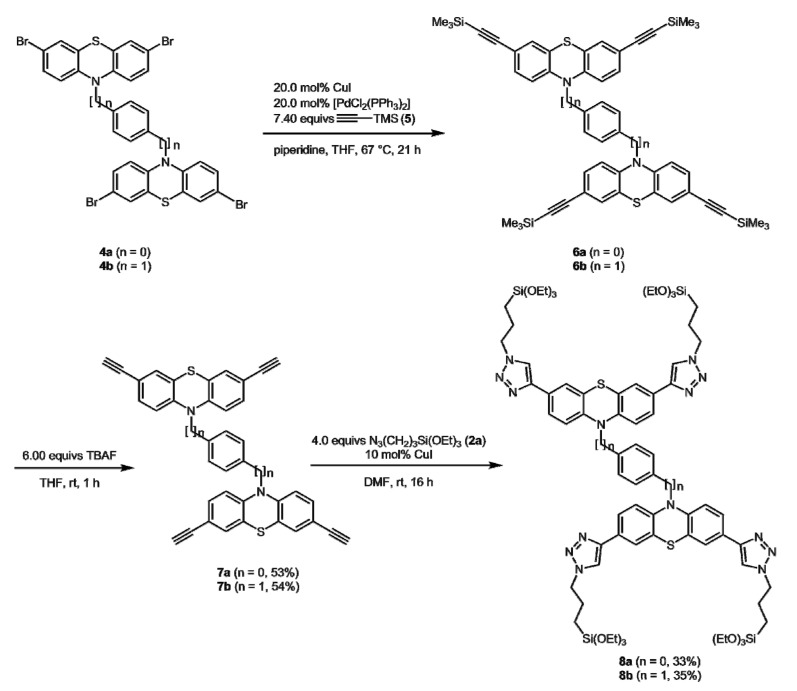
CuAAC synthesis of triazolyl conjugated tetrakis(triethoxylsilylpropyltriazolyl) substituted diphenothiazine dumbbells **8**.

For the synthesis of the conjugated and nonconjugated diphenothiazine dumbbells **8** decorated with conjugated triazoles bearing triethoxylsilyl propyl groups, first the tetrakis(ethynyl) compounds **7** were synthesized by Sonogashira alkynylation of the tetra-bromo derivatives **4** with (trimethylsilyl)acetylene (**5**) followed by desilylation of the crude product **6** (Scheme 2). Similarly to the formation of the precursors **3** the conjugated and nonconjugated diphenothiazine dumbbells **8** bearing conjugated triazoles with tri-ethoxylsilyl propyl groups were synthesized by CuAAC with (3-azidopropyl)triethoxy-silane (**2a**) in moderate yield. The structures of compounds **8** were unambiguously assigned by IR, ^1^H- and ^13^C-NMR spectroscopy and mass spectrometry.

### 2.2. Electronic Properties of the Triazolyl Conjugated (Oligo)Phenothiazines

The ground and excited state electronic properties of the triazolyl conjugated (oligo)-phenothiazines **3** and **8** was studied by cyclic voltammetry, absorption and emission spectroscopy (Table 1).

**Figure 1:**
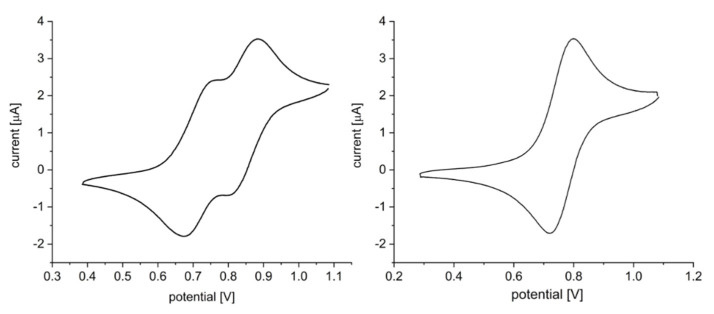
Cyclic voltammograms of triazolyl conjugated diphenothiazines **8a** (left) and **8b** (right) (recorded in dichloromethane at *T* = 298 K, *ν* = 100 mVs^−1^, elektrolyte: [Bu_4_N][PF_6_], Pt working electrode, Pt counter electrode, Ag/AgCl reference electrode, referenced to *E_1/2_^0/+1^*(Me_10_Fc/Me_10_Fc^+^) = −95 mV).

All (oligo)phenothiazines triazolyl-conjugated (oligo)phenothiazines **3** and **8** possess fully chemically reversible first oxidation potentials between 650 and 800 mV (Table 1, Figure 1). Upon direct comparison of the first oxidation potentials of *N*-methyl phenothiazine (*E_1/2_^0/+1^* = 770 mV) or *N*-hexyl phenothiazine (*E_1/2_^0/+1^* = 730 mV) [32] to mono phenothiazines **3a** (*E_1/2_^0/+1^* = 750 mV) and **3b** (*E_1/2_^0/+1^* = 720 mV), and **3d** (*E_1/2_^0/+1^* = 730 mV) and **3e** (*E_1/2_^0/+1^* = 700 mV) it becomes apparent that the triazole electronically exerts a weak electron-releasing effect [22]. The directly conjugated dyads **3c** and **3f** are more electron-rich, with lowest first oxidations at *E_1/2_^0/+1^* = 650 mV, and reveal two consecutive oxidations se-parated by 140 to 170 mV arising from diphenothiazinyl coupling [36]. Likewise, the *N*-bridged conjugated dyad **8a** displays a similiar behavior of a coupled redox system consisting of two electrophore termini with a separation of the two one-electron oxidations of 180 mV (Figure 1 left). In contrast, the deconjugated dyad **8b** only shows a single reversible first oxidation, which occurs simultaneously at both electrophore moieties, i.e., without any electronic coupling (Figure 1 right).

**Figure 2:**
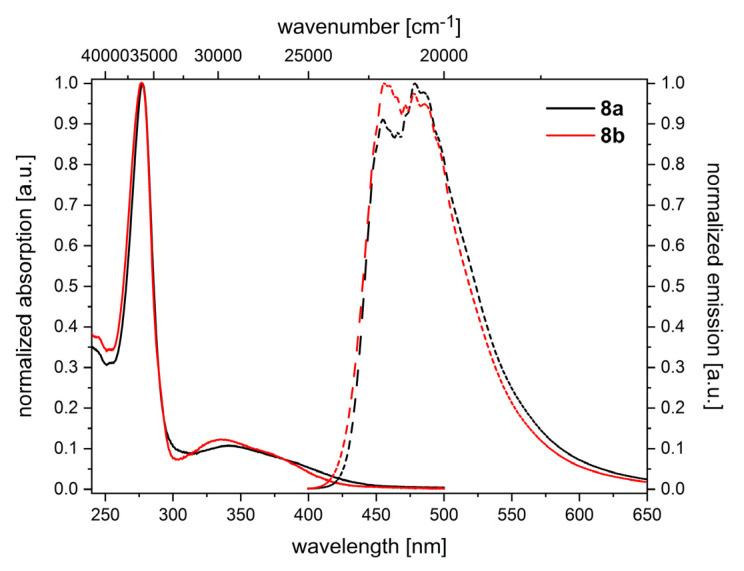
Absorption (solid lines) and emission (dashed lines) spectra of triazolyl conjugated diphenothiazines **8a** (black) and **8b** (red) (recorded in dichloromethane at *T* = 298 K with *c*(**8**) = 10^−3^ m).

Electron absorption and emission spectroscopy clearly show that all compounds in this study are luminescent with typical huge phenothiazine Stokes shifts [36]. More specifically for the series **3**, the absorption in the UV region is accompanied by a blue to greenish blue fluorescence for all representatives. The emission maxima lie between 446 and 470 nm. The Stokes shifts in a range from $\Delta \tilde{\nu }$ = 5700 to 9200 cm^−1^ are caused by significant geometrical changes upon excitation from a highly non-planar ground state to a largely planarized excited state [37]. Although showing a different mode of ligation the dyads **8** possess very similar emission maxima *λ*_em,max_ at 455 (**8a**) and 456 nm (**8b**), indicating that the major contribution of the luminescence stems from the bis(triazolyl)phenothiazine structural element (Figure 2). Essentially the same reasoning for huge Stokes shifts arising from planarization of the excited state upon UV excitation also holds true for this series.

### 2.3. Silica Hybrid Materials from Selected Triazolyl Conjugated (Oligo)Phenothiazines ***3b-d***

**Scheme 3:**
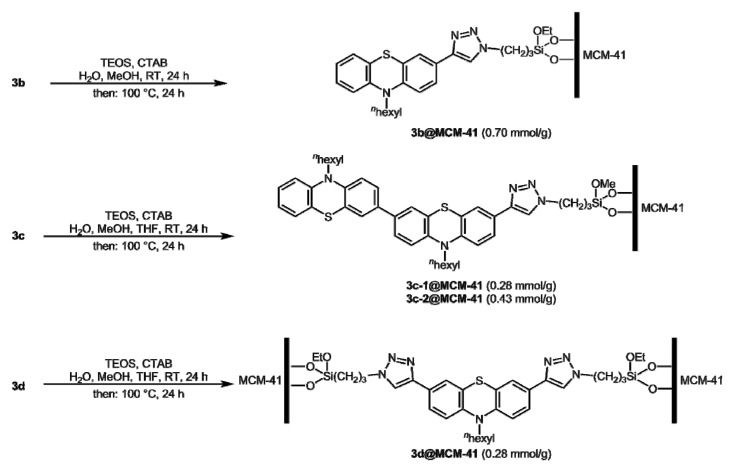
In situ synthesis of selected triazolyl conjugated (oligo)phenothiazine silica hybrid materials **3b**–**d@MCM-41**.

Encouraged by our successful synthesis of fluorophore-containing silica hybrid materials from triazolyl ligated precursors [26,27] we reasoned this type of precursor ligation could be employ for embedding electrophores in the sense of an in-situ synthesis of meso-porous hybrid materials. Exemplarily the three triazolyl conjugated phenothiazine deri-vatives **3b**, **3c**, and **3d** were chosen as substrates for the material synthesis. The in situ synthesis of silica hybrid materials was performed by first stirring an aqueous mixture of the surfactant cetyltrimethylammonium bromide (CTAB), ethylamine, precursor **3b**, methanol (and THF for precursors **3c** and **3d**), and tetraethylorthosilicate (TEOS) at room temp for 24 h followed by heating to 100 °C for another 24 h (Scheme 3). After extraction of CTAB the powdery materials **3b**–**d@MCM-41** were obtained and the loadings of the electrophores **3b**–**d** were determined by combustion analysis.

### 2.4. Structural Characterization of Silica Hybrid Materials **3b–d@MCM-41**

First encouraging information on the silica hybrid materials **3b**–**d@MCM-41** is provided by the phenothiazine content that was determined by combustion analysis (Table 2). Expectedly, the smaller the precursor **3b**–**d** the higher is the phenothiazine weight percentage in the derived hybrid materials **3b**–**d@MCM-41**. Interestingly, the employed precursor concentration also affects phenothiazine loading as seen by the phenothiazine content of the samples **3c-1@MCM-41** and **3c-2@MCM-41**.

Powder X-ray diffraction (XRD) was employed as a routine method to characterize the ordering of the mesostructure (Table 2, for a detailed discussion of the powder XRD data, see Supplementary Materials). The XRD patterns of the hybrid materials **3b**–**d@MCM-41** show the typical reflections of the [100] plane of hexagonal symmetry at 2θ = 2.0 to 2.1° corresponding to *d*-values between 4.20 to 4.50 nm. Additionally, overlapping broad bands can be observed in the 2θ rage of 3.4 to 4.8°, which can be assigned to the diffraction of the 110 and 200 planes of the hexagonally symmetric lattice of the MCM-41 type materials [38]. Therefore, periodic arrangement of pores in the materials are present, although these diffraction peaks are neither very sharp nor intense. This decreased intensity and broadening of the diffraction peaks disturbs the contrast between the framework and the pore channels [39,40], which can be attributed to structural distortions caused by the stiffness of precursor materials **3** in cylindrical channels [41]. In comparison to the cell parameter *a_0_* = 4.96 nm of pure silica MCM-41 [30] the cell parameters *a_0_* = 4.85–5.20 nm fall into a similar range. While material **3b@MCM-41** possesses a slightly shrunk lattice, the lattices of **3c,d@MCM-41** formed from precursors **3c** and **3d** reveal a slightly expanded lattice. Caused by the size and geometry, the mesostructure of material **3d@MCM-41** is similar to a material with a wormhole-like pore system (or HMS silica phases) [42].

Nitrogen physisorption measurements provide information on the pore structure and surface parameter of the hybrid materials **3b-d@MCM-41**. Textural parameters derived from isotherms and the pore distribution curves (see Supplementary Materials) are collected in Table 2. The isotherms and their pore distribution curves clearly support the mesoporous structure of the materials **3b-d@MCM-41**. The data of the two samples of **3c@MCM-41** with varying phenothiazine content expectedly give different surface areas and pore volumes.

**Figure 3:**
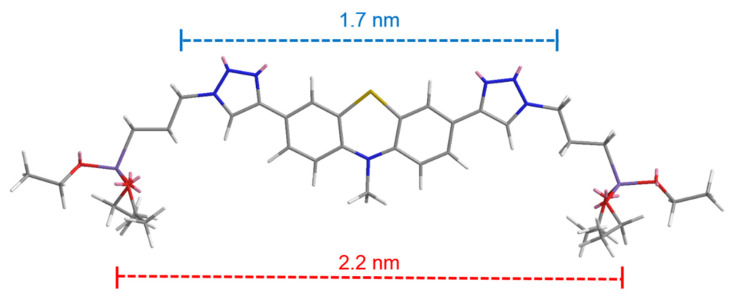
MM2-optimized stretched conformer of precursor **3d** highlighting the intramolecular Si-Si distance of 2.2 nm and the rigid core with an extension of 1.7 nm.

The estimated wall thickness of **3d@MCM-41** of 2.76 nm is rather high compared to pure silica MCM-41 obtained under similar conditions as well as to other phenothiazine containing hybrid materials anchored on the internal surface [28,30]. Due to the relatively large dimensions of precursor **3d** (Figure 3 highlights the rigid region and the maximum intramolecular Si-Si distance of the MM2-calculated expanded conformer) the ligation distributes this precursor also inside the channel walls. With an average Si-O bond length of 1.6 Å and the size of precursor **3d**, two putative scenarios can be envisioned: First, the molecules are embedded inside the walls of the material, or second, the molecules are placed outside of the wall stretching across the pore with both silicon atoms as anchoring sites.

**Figure 4:**
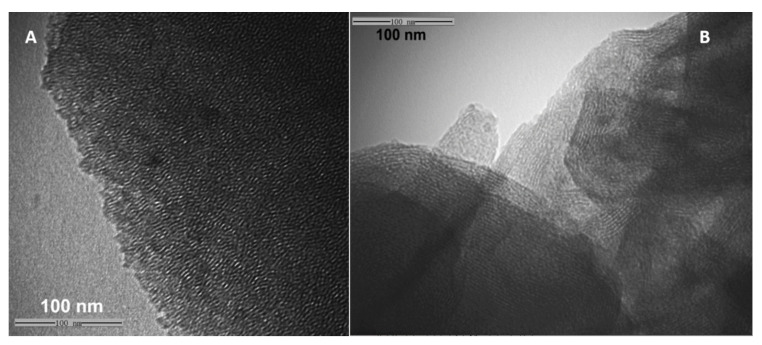
TEM images of **3d@MCM-41**. (**A**) Along the channel direction. (**B**) Perpendicular to the channel direction.

Furthermore, transmission electron microscopy (TEM) supports the mesoporous structure of the hybrid materials **3b**–**d@MCM-41** (for details, see Supplementary Materials), additionally revealing distortions from ideal parallel orientation of the channels caused by the large magnitude of some precursors even leading to worm-hole like morphology for the material **3d@MCM-41** formed from the largest precursor **3d** in this series (Figure 4).

Spectroscopic characterization of the hybrid materials **3b-d@MCM-41** by FT-IR, ^13^C CP-MAS, and ^29^Si CP-MAS NMR spectroscopy additionally supports the molecular structural features of the materials (for details, see Supplementary Materials). In particular, ^13^C CP-MAS NMR spectra of the materials **3b-d@MCM-41** in comparison the solution spectra of the corresponding precursors **3b-d** manifests the unambiguous incorporation of the organic moieties within the materials without disintegration during the hydrothermal synthesis and the acidic extraction process. Finally, ^29^Si CP-MAS NMR spectroscopy outlines the presence of organic/inorganic moieties as basic structural units constituting the hybrid mesoporous materials.

### 2.5. Optical Properties and Chemical Oxidation of Hybrid Materials **3b–d@MCM-41**

As already seen from the optical properties of the precursors **3b**–**d** the effect of conjugated triazolyl moieties in the hybrid materials **3b**–**d@MCM-41** not only affects the mode of ligation. Interestingly, some absorption and emission characteristics, although obtained from solution data for the precursors and in reflectance mode for the materials, are very similar for precursors and materials, but some also distinctly differ from each other (Table 3, for spectra, see Supplementary Materials).

Hybrid material **3b@MCM-41**, which is a yellow powder, and its precursor **3b**, exhibit very similar absorption and emission characteristics: two absorption bands at around 260 and 320 nm and one emission band at around 451 nm. This accounts for the absence of any interaction of the chromophore with the framework of the material. Essentially, also the chromophores are equally distributed in the material without any interchromophore interactions. The remarkably large Stokes shift (~9000 cm^−1^) demonstrating a significant geometrical change upon the excitation from a highly non-planar ground state to largely planarized excited state is typical for phenothiazine derivatives [37]. The chromophores contain triazole moieties that can be protonated by sufficiently strong acids. Upon protonation of hybrid material **3b@MCM-41-H^+^** with trifluoroacetic acid a redshift of the longest wavelength absorption band from 318 to 364 nm can be observed (for details, see Supplementary Materials). The triazole protonation causes an increase of the substituents electron withdrawing character to a −M substituent which plausibly diminishes the HOMO-LUMO gap and explains the observed halochromicity.

The color of hybrid material **3c@MCM-41** is dark yellow and the red-shifted color can be explained with the large π-system of the constituting biphenothiazine chromophore, as also supported by the bathochromic shift of the absorption bands in comparison to hybrid material **3b@MCM-41**. This extended π-system also causes a red shift of the emission bands at 484 nm (**3c-1@MCM-41**) and 500 nm (**3c-2@MCM-41**). Interestingly, the higher chromophore loading of material **3c-2@MCM-41** not only causes a pronounced red shift but even a more intense blue emission upon eyesight.

The adsorption and emission spectra of material **3d@MCM-41** containing a phenothiazine with two ligation positions possess in addition to two absorption bands at 270 and 330 nm typical for phenothiazines and a small shoulder appears at around 360 nm. Interestingly, the emission spectrum of this hybrid material displays dual emission with maxima around 445 and 390 nm, which are absent in the solution spectrum of precursor **3d**. Considering that the molecules are uniformly distributed inside the silica framework, this dual emission is likely caused by conformational biases due to restricted molecular rotation and relaxation.

**Scheme 4:**
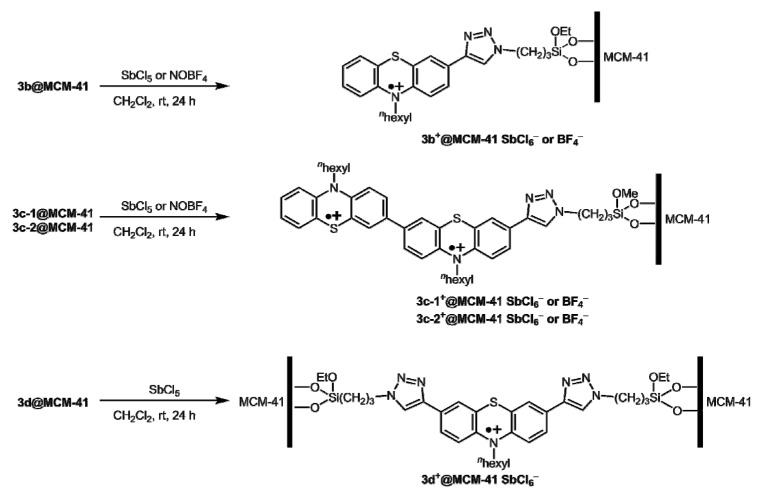
Oxidation of the silica hybrid materials **3b-d@MCM-41** with single electron oxidants.

Encouraged by the apparent stability of radical cations of the phenothiazine containing precursors **3** as shown by reversible electrochemical oxidation under the conditions of cyclic voltammetry we decided to chemically oxidize the silica hybrid materials with one electron oxidants, such as antimony pentachloride and NOBF_4_, in dichloromethane suspension at room temp (Scheme 4).

The color of the materials **3b**–**d@MCM-41** immediately changes from yellow to dark green-blue after addition of the oxidant. In the absorption spectra of the suspensions, radical cation absorption signatures at low energy are discernable. However, the stronger oxidation potential of SbCl_5_ not only causes differences in the absorption spectra due to counter ion effects, but also due to more complete oxidation more intense absorption bands of the resulting oxidized hybrid materials **3b**–**d^+^@MCM-41 X**^−^ are found (for spectra and details, see Supplementary Materials). Interestingly, the suspension of cationic material **3b^+^@MCM-41 BF_4_**^−^ also shows a protochromic behavior as the pristine materials **3b@MCM-41** (for spectra, see Supplementary Materials). In the solid state, all oxidized hybrid materials are deeply colored, mostly dark green to black. Remarkably also for material **3d@MCM-41** with phenothiazines embedded in the silica walls, these electrophores are reached by the chemical oxidant, as also observed by others [43]. Another interesting feature is that the obtained oxidized materials are obviously quite stable under a nitrogen atmosphere at ambient temperature for several weeks due to absence of any bleaching of the radical cations.

**Figure 5:**
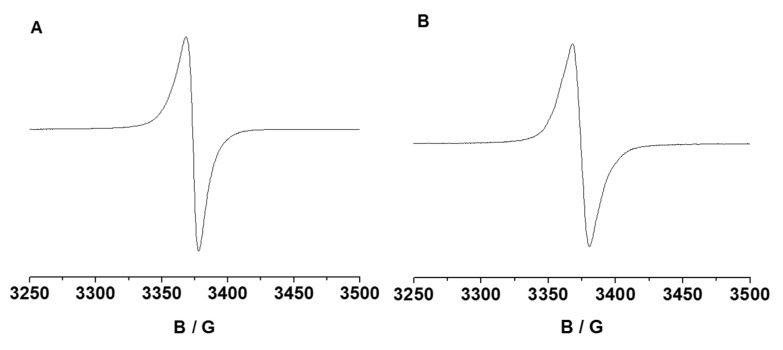
Q-band EPR spectra of (**A**) **3b^+^@MCM-41 SbCl_6_**^−^ and (**B**) **3d^+^@MCM-41 SbCl_6_**^−^ (recorded at *T* = 297 K).

Finally, electron paramagnetic resonance (EPR) spectra were recorded for the oxidized hybrid materials **3b^+^@MCM-41 SbCl_6_**^−^ and **3d^+^@MCM-41 SbCl_6_**^−^ to provide further evidence of the presence of stable phenothiazine radical cations in the hybrid materials (Figure 5). The g-values of these two samples are 2.0054 and 2.0050, respectively. Although, the EPR signals are not well resolved and hyperfine coupling constants and an-isotropic g-tensors cannot be determined, the high symmetry of the EPR spectra indicates that the phenothiazine radicals find themselves in a quite isotropic environment in the hybrid materials. This remarkable chemical stability of phenothiazinyl radical cations in mesoporous silica hybrid materials makes them suitable for studying radical processes in mesoporous environments [44].

## 3. Materials and Methods

### 3.1. General Procedure (GP1) for CuAAC Synthesis of 1-(3-(Triethoxysilyl)propyl)-1,2,3-Triazol-4-yl Substituted (Oligo)phenothiazine Precursors ***3***

To a well-stirred mixture phenothiazinyl alkyne **1a**–**f** (1.0 equiv), placed in a Schlenk tube with a magnetic stir bar and dissolved in dry DMF, copper iodide (10 mol%) was added (For details, see Table 4). Then, 3-(triethoxysilyl)propylazide (**2a**) or 3-(trimethoxy-silyl)propylazide (**2b**) (1.0 equiv per CC triple bond) was added, the reaction tube was sealed and the suspension was stirred under nitrogen at room temp for 16 h. The reaction mixture was filtered through a short plug of Celite^®^ and washed with dichloromethane. The solvents were removed in vacuo and the product was dried in vacuo for 14 h.

#### 3.1.1. 10-Methyl-3-(1-(3-(triethoxysilyl)propyl)-1*H*-1,2,3-triazole-4-yl)-10*H*-phenothiazine (**3a**)

According to GP1 the triazole **3a** (193 mg, 95%) was isolated as a brown oil. ^1^H-NMR (CD_2_Cl_2_, 500 MHz): *δ* 0.62 (t, *J* = 8.0 Hz, 2 H), 1.20 (t, *J* = 7.0 Hz, 9 H), 2.02 (m, 2 H), 3.39 (s, 3 H), 3.80 (q, *J* = 7.0 Hz, 6 H), 4.36 (t, *J* = 7.0 Hz, 2 H), 6.87 (m, 2 H), 6.94 (dt, *J* = 7.5 Hz, *J* = 1.0 Hz, 1 H), 7.14 (dd, *J* = 1.0 Hz, *J* = 7.5 Hz, 1 H), 7.19 (m, 1 H), 7.58 (d, *J* = 2.0 Hz, 1 H), 7.64 (dd, *J* = 2.0 Hz, *J* = 8.5 Hz, 1 H), 7.73 (s, 1 H). ^13^C-NMR (CD_2_Cl_2_, 125 MHz): *δ* 7.1 (CH_2_), 17.8 (CH_3_), 23.9 (CH_2_), 34.9 (CH_3_), 52.2 (CH_2_), 58.1 (CH_2_), 113.8 (CH), 113.8 (CH), 118.8 (CH), 122.1 (CH), 122.5 (C_quat_), 123.4 (C_quat_), 123.6 (CH), 124.3 (CH), 125.1 (C_quat_), 126.7 (CH), 127.2 (CH), 128.2 (C_quat_), 145.1 (C_quat_), 145.2 (C_quat_). UV/Vis (CH_2_Cl_2_) *λ*_max_ (ε): 266 (49200), 319 nm (9950). IR (film): $\tilde{\nu }$ = 2889, 2098, 1467, 1334, 1260, 1103, 954, 751, 698, 561, 534 cm^−1^. MS (FAB^+^) *m/z* (%): 484.3 (100, M^+^), 439.2 (18, M^+^−C_2_H_5_O), 422.2 (17), 376.3 (42, M^+^− CH_2_Si(OC_2_H_5_)_3_).

#### 3.1.2. 10-Hexyl-3-(1-(3-(triethoxysilyl)propyl)-1*H*-1,2,3-triazole-4-yl)-10*H*-phenothiazine (**3b**)

According to GP1 the triazole **3b** (228 mg, 97%) was isolated as a brown oil. ^1^H-NMR (CD_2_Cl_2_, 500 MHz): *δ* 0.62 (t, *J* = 8.0 Hz, 2 H), 0.88 (t, *J* = 7.0 Hz, 3 H), 1.20 (t, *J* = 7.0 Hz, 9 H), 1.31 (m, 4 H), 1.44 (m, 2 H), 1.80 (m, 2 H), 2.03 (m, 2 H), 3.80 (q, *J* = 7.0 Hz, 6 H), 3.86 (t, *J* = 7.5 Hz, 2 H), 4.36 (t, *J* = 7.5 Hz, 2 H), 7.16 (m, 2 H), 7.45 (m, 2 H), 7.55 (d, *J* = 2.0 Hz, 1 H), 7.62 (dd, *J* = 8.5 Hz, *J* = 2.0 Hz, 1 H), 7.72 (s, 1 H), 7.87 (m, 1 H). ^13^C-NMR (CD_2_Cl_2_, 125 MHz): *δ* 7.1 (CH_2_), 13.4 (CH_3_), 17.8 (CH_3_), 22.3 (CH_2_), 23.9 (CH_2_), 26.2 (CH_2_), 26.5 (CH_2_), 31.1 (CH_2_), 41.7 (CH_2_), 52.2 (CH_2_), 58.1 (CH_2_), 115.1 (CH), 115.2 (CH), 118.8 (CH), 122.0 (C_quat_), 123.8 (C_quat_), 124.2 (CH), 127.0 (C_quat_), 128.1 (CH), 128.2 (C_quat_), 131.3 (CH), 131.6 (CH), 131.7 (CH), 144.6 (C_quat_), 144.7 (C_quat_). UV/Vis (CH_2_Cl_2_) *λ*_max_ (ε): 268 (55400), 318 nm (14250). IR (film): $\tilde{\nu }$ = 2973, 2927, 1719, 1655, 1579, 1544, 1459, 1390, 1340, 1290, 1252, 1166, 1102, 1080, 957, 793, 750, 726, 698, 560, 528 cm^−1^. MS (FAB^+^) *m/z* (%): 554.3 (100, M^+^), 509.3 (12, M^+^− C_2_H_5_O), 483.2 (6, M^+^− C_5_H_11_), 422.2 (12), 376.3 (24, M^+^− CH_2_Si(OC_2_H_5_)_3_).

#### 3.1.3. 10,10’-Dihexyl-7-(1-(3-(trimethoxysilyl)propyl)-1*H*-1,2,3-triazole-4-yl)-10*H*,10’*H*-3,3’-biphenothiazine (**3c**)

According to GP1 the triazole **3c** (970 mg, 98%) was isolated as a brown oil. ^1^H-NMR (CD_2_Cl_2_, 500 MHz): *δ* 0.65 (t, *J* = 8.0 Hz, 2 H), 0.89 (m, 6 H), 1.31 (m, 8 H), 1.44 (m, 4 H), 1.79 (m, 4 H), 2.04 (m, 2 H), 3.57 (s, 9 H), 3.84 (m, 4 H), 4.36 (t, *J* = 7.0 Hz, 2 H), 6.89 (m, 5 H), 7.15 (m, 2 H), 7.32 (m, 4 H), 7.60 (m, s, 1 H), 7.64 (m, 1 H), 7.74 (s, 1 H). ^13^C-NMR (CD_2_Cl_2_, 125 MHz): *δ* 5.8 (CH_2_), 13.5 (CH_3_), 22.3 (CH_2_), 23.6 (CH_2_), 26.2 (CH_2_), 26.3 (CH_2_), 26.4 (CH_2_), 31.1 (CH_2_), 47.0 (CH_2_), 47.1 (CH_2_), 50.1 (CH_3_), 52.1 (CH_2_), 115.0 (CH), 115.1 (CH), 118.8 (CH), 121.9 (CH), 123.8 (CH), 124.1 (C_quat_), 124.2 (CH), 124.4 (CH), 124.6 (C_quat_), 124.7 (CH), 124.8 (CH), 124.9 (C_quat_), 126.9 (CH), 133.5 (C_quat_), 133.7 (C_quat_), 143.4 (C_quat_), 143.8 (C_quat_), 144.3 (C_quat_), 144.7 (C_quat_), 146.1 (C_quat_). UV/Vis (CH_2_Cl_2_) *λ*_max_ (ε): 278 (43200), 328 (14600), 367 nm (12300). IR (film): $\tilde{\nu }$ = 2926, 2852, 1672, 1577, 1508, 1458, 1376, 1332, 1251, 1191, 1082, 874, 807, 764 cm^−1^. MS (MALDI) calcd. for [C_44_H_55_N_5_O_3_S_2_Si]^+^ *m/z*: 793.3; found: 793.3 (M^+^).

#### 3.1.4. 10-Methyl-3,7-bis(1-(3-(triethoxysilyl)propyl)-1*H*-1,2,3-triazole-4-yl)-10*H*-phenothiazine (**3d**)

According to GP1 the bistriazole **3d** (140 mg, 88%) was isolated as a brown oil. ^1^H-NMR (CD_2_Cl_2_, 500 MHz): *δ* 0.62 (t, *J* = 8.0 Hz, 4 H), 1.20 (t, *J* = 7.0 Hz, 18 H), 2.03 (m, 4 H), 3.42 (s, 3 H), 3.80 (q, *J* = 7.0 Hz, 12 H), 4.37 (t, *J* = 7.5 Hz, 4 H), 6.90 (m, 1 H), 7.45 (m, 2 H), 7.60 (m, 2 H), 7.74 (m, 1 H), 7.87 (m, 2 H). ^13^C-NMR (CD_2_Cl_2_, 125 MHz): *δ* 7.1 (CH_2_), 17.8 (CH_3_), 23.8 (CH_2_), 35.0 (CH_3_), 52.2 (CH_2_), 58.1 (CH_2_), 114.0 (CH), 118.8 (CH), 123.0 (C_quat_), 123.7 (CH), 124.4 (CH), 125.3 (C_quat_), 128.2 (CH), 131.7 (CH), 144.8 (C_quat_), 146.1 (C_quat_). UV/Vis (CH_2_Cl_2_) *λ*_max_ (ε): 276 (37800), 334 nm (7200). IR (film): $\tilde{\nu }$ = 2974, 1457, 1392, 1337, 1263, 1079, 957, 787, 698, 560 cm^−1^. MS (MALDI) calcd. for [C_35_H_53_N_7_O_6_SSi_2_]^+^ *m/z*: 756.335, found: 756.297 (M^+^).

#### 3.1.5. 10-Hexyl-3,7-bis(1-(3-(triethoxysilyl)propyl)-1*H*-1,2,3-triazole-4-yl)-10*H*-phenothiazine (**3e**)

According to GP1 the bistriazole **3e** (625 mg, 93%) was isolated as a brown oil. ^1^H-NMR (CD_2_Cl_2_, 500 MHz): *δ* 0.62 (t, *J* = 7.7 Hz, 4 H), 0.88 (t, *J* = 7.0 Hz, 3 H), 1.20 (t, *J* = 7.0 Hz, 18 H), 1.33 (m, 4 H), 1.46 (m, 2 H), 1.83 (m, 2 H), 2.03 (m, 4 H), 3.80 (q, *J* = 7.0 Hz, 12 H), 3.89 (t, *J* = 7.3 Hz, 2 H), 4.37 (t, *J* = 7.3 Hz, 4 H), 6.93 (d, *J* = 8.5 Hz, 2 H), 7.57 (d, *J* = 2.0 Hz, 2 H), 7.63 (dd, *J* = 8.5 Hz, *J* = 2.0 Hz, 2 H), 7.73 (s, 2 H). ^13^C-NMR (CD_2_Cl_2_, 125 MHz): *δ* 7.0 (CH_2_), 13.4 (CH_3_), 17.8 (CH_3_), 22.3 (CH_2_), 23.9 (CH_2_), 26.2 (CH_2_), 26.4 (CH_2_), 31.1 (CH_2_), 41.7 (CH_2_), 52.2 (CH_2_), 58.1 (CH_2_), 115.2 (CH), 123.8 (CH), 124.2 (C_quat_), 124.3 (CH), 125.1 (CH), 144.2 (C_quat_), 148.1 (C_quat_). UV/Vis (CH_2_Cl_2_) *λ*_max_ (ε): 276 (54500), 335 nm (10100). IR (film): $\tilde{\nu }$ = 2974, 2928, 1871, 1736, 1719, 1655, 1618, 1560, 1458, 1400, 1365, 1296, 1257, 1166, 1079, 956, 789 cm^−1^. MS (MALDI) calcd. for [C_40_H_63_N_7_O_6_SSi_2_]^+^ *m/z*: 825.4; found: 825.4 (M^+^).

#### 3.1.6. 10,10’Dihexyl-7,7’-bis(1-(3-(trimethoxysilyl)propyl)-1*H*-1,2,3-triazole-4-yl)-10*H*,10’*H*-3,3’-biphenothiazine (**3f**)

According to GP1 the bistriazole **3f** (90 mg, 92%) was isolated as a brown oil. ^1^H-NMR (CD_2_Cl_2_, 500 MHz): *δ* 0.64 (t, *J* = 8.0 Hz, 4 H), 0.88 (t, *J* = 7.0 Hz, 6 H), 1.33 (m, 8 H), 1.46 (m, 4 H), 1.82 (m, 4 H), 2.02 (m, 4 H), 3.55 (s, 18 H), 3.88 (t, *J* = 7.0 Hz, 4 H), 4.36 (t, *J* = 7.0 Hz, 4 H), 6.93 (d, *J* = 3.5 Hz, 2 H), 7.32 (d, *J* = 2.0 Hz, 2 H), 7.35 (dd, *J* = 8.0 Hz, *J* = 2.0 Hz, 2 H), 7.56 (d, *J* = 2.0 Hz, 2 H), 7.62 (dd, *J* = 8.0 Hz, *J* = 2.0 Hz, 2 H), 7.72 (s, 2 H). ^13^C-NMR (CD_2_Cl_2_, 125 MHz): *δ* 5.8 (CH_2_), 13.4 (CH_3_), 22.3 (CH_2_), 23.7 (CH_2_), 26.2 (CH_2_), 26.4 (CH_2_), 31.1 (CH_3_), 47.7 (CH_2_), 52.1 (CH_2_), 115.1 (CH), 115.2 (CH), 118.7 (CH), 122.6 (C_quat_), 122.8 (C_quat_), 123.2 (C_quat_), 123.4 (C_quat_), 123.9 (CH), 124.2 (CH), 124.3 (CH), 124.8 (CH), 131.6 (C_quat_), 142.8 (C_quat_), 144.1 (C_quat_). UV/Vis (CH_2_Cl_2_) *λ*_max_ (ε): 278 (50700), 336 nm (16100). IR (film): $\tilde{\nu }$ = 2926, 1719, 1611, 1560, 1459, 1400, 1400, 1340, 1254, 1191, 1079, 874, 803 cm^−1^. MS (MALDI) calcd. For [C_52_H_70_N_8_O_6_S_2_Si_2_]^+^ *m/z*: 1022.44; found: 1022.5 (M^+^).

### 3.2. 1,4-Di(10H-phenothiazin-10-yl)benzene 

In an oven-dried Schlenk tube with a magnetic stir bar were placed 10*H*-phenothiazine (3.99 g, 20.0 mmol), 1,4-diiodobenzene (3.30 g, 10.0 mmol), bis(dibenzylideneacetone)palladium(0) (350 mg, 6.00 mol%), tri(*tert*-butyl)phosphonium tetrafluoroborate (290 mg, 10.0 mol%), sodium *tert*-butanolate (2.21 g, 23.0 mmol), and dry 1,4-dioxane (80.0 mL). The dark solution was deaerated by a constant stream of nitrogen via syringe for 10 min. Then, the reaction mixture was heated to reflux for 16 h. After cooling to room temp the solvents were removed in vacuo and the residue was absorbed on celite^®^ and purified by column chromatography on silica gel (*n*-hexane/ethyl acetate (9:1 → 1:1) to give 1,4-di(10*H*-phenothiazin-10-yl)benzene [45,46] (4.44 g, 94%) as a light beige powder, Mp 258–260 °C. *R_f_* (*n*-hexane/acetone 9:1): 0.30. ^1^H-NMR (300 MHz, di-methylsulfoxide-d_6_): *δ* 6.55 (dd, ^3^*J* = 8.1 Hz, ^4^*J* = 1.1 Hz, 4 H), 6.97 (ddd, ^3^*J* = 7.5, 7.8 Hz, ^4^*J* = 1.1 Hz, 4 H), 7.09 (ddd, ^3^*J* = 8.1 Hz, 7.8 Hz, ^4^*J* = 1.5 Hz, 4 H), 7.19 (dd, ^3^*J* = 7.5 Hz, ^4^*J* = 1.5 Hz, 4 H), 7.53 (s, 4 H). ^13^C-NMR (500 MHz, dimethylsulfoxide-d_6_): *δ* 118.5 (CH), 122.3 (C_quat_), 123.8 (CH), 127.4 (CH), 127.9 (CH), 130.4 (CH), 140.3 (C_quat_), 143.6 (C_quat_). MS (EI) *m/z*: 472 (5, [M]^+^), 352 (93, C_23_H_16_N_2_S]^+^). IR $\tilde{\nu }$ [cm^−1^]: 3061 (w), 3032 (vw), 1884 (vw), 1591 (m), 1556 (m), 1501 (s), 1483 (w), 1460 (vs), 1443 (s), 1406 (vw), 1369 (vw), 1304 (s), 1279 (w), 1254 (s), 1236 (s), 1184 (w), 1165 (vw), 1155 (vw), 1126 (m), 1084 (m), 1042 (s), 1017 (m), 961 (w), 930 (s), 840 (m), 739 (vs), 723 (s), 712 (s), 700 (vw), 664 (m), 627 (vs). Anal. calcd. for C_30_H_20_N_2_S_2_ [472.6]: C 76.24, H 4.27, N 5.93, S 13.57; Found: C 76.41, H 4.19, N 5.76, S 13.50.

### 3.3. 1,4-Bis(3,7-dibromo-10H-phenothiazin-10-yl)benzene *(**4a**)*

In an oven-dried 250 mL two-necked round bottom flask with a magnetic stir bar were placed 1,4-di(10*H*-phenothiazin-10-yl)benzene (1.35 g, 2.86 mm) and glacial acetic acid (823 mL). A solution of elementary bromine (740 μL, 14.3 mmol) in acetic acid (114 mL) was added dropwise over an hour to the vigorously stirred solution at room temp. The dark brown solution was then stirred at room temp for 16 h. Then a saturated aqueous solution of sodium sulfite (720 mg, 5.71 mmol) was added and the solution was stirred at room temp for 3 h. The precipitate was collected by suction and was washed three times with methanol. Then the product was dried under vacuo to give compound **4a** (2.10 g, 93%) as a light yellow powder, Mp 356–358 °C. *R_f_* (*n*-hexane/dichloromethane 9:1): 0.15. ^1^H-NMR (300 MHz, dimethylsulfoxide-d_6_): *δ* 6.33 (d, ^3^*J* = 8.8 Hz, 4 H), 7.21 (dd, ^3^*J* = 8.8 Hz, ^4^*J* = 2.3 Hz, 4 H), 7.39 (d, ^4^*J* = 2.3 Hz, 4 H), 7.62 (s, 4 H). ^13^C-NMR (75 MHz, dimethylsulfoxide-d_6_): *δ* 114.8 (C_quat_), 119.0 (CH), 122.6 (C_quat_), 128.8 (CH), 130.3 (CH), 131.6 (CH), 139.6 (C_quat_), 142.2 (C_quat_). MS (EI) *m/z*: 792 (15, [M^81^Br_4_]^+^), 790 (47, [M^81^Br_3_^79^Br]^+^), 788 (68, [M^81^Br_2_^79^Br_2_]^+^), 786 (44, [M^81^Br_3_^79^Br]^+^), 784 (6, [M^79^Br_4_]^+^), 711 (15, [C_30_H_16_^81^Br_3_N_2_S_2_]^+^), 709 (22, [C_30_H_16_^81^Br_2_^79^BrN_2_S_2_]^+^), 707 (20, [C_30_H_16_^81^Br^79^Br_2_N_2_S_2_]^+^), 705 (6, [C_30_H_16_^79^Br_3_N_2_S_2_]^+^), 630 (9, [C_30_H_16_^81^Br_2_N_2_S_2_]^+^), 628 (12, [C_30_H_16_^81^Br^79^BrN_2_S_2_]^+^), 626 (5, [C_30_H_16_^79^Br_2_N_2_S_2_]^+^), 358 (32, [C_12_H_6_^81^Br_2_NS]^+^), 356 (65, [C_12_H_6_^81^Br^79^BrNS]^+^), 354 (39, [C_12_H_6_^79^Br_2_NS]^+^), 272 (43, [C_18_H_10_NS]^+^), 196 (100, [C_12_H_6_NS]^+^). IR $\tilde{\nu }$ [cm^−1^]: 3917 (vw), 2959 (vw), 2857 (vw), 2332 (w), 2305 (vw), 1582 (m), 1504 (s), 1481 (m), 1452 (vs), 1406 (w), 1379 (m), 1346 (vw), 1308 (s), 1279 (w), 1265 (w), 1254 (m), 1242 (m), 1200 (vw), 1186 (vw), 1152 (vw), 1123 (vw), 1096 (m), 1078 (w), 1067 (vw), 1018 (w), 951 (vw), 920 (w), 866 (m), 856 (m), 839 (m), 820 (vw), 797 (vs), 754 (m), 735 (w), 716 (vw), 685 (w), 669 (w). Anal. calcd. for C_30_H_16_N_2_S_2_Br_4_ [788.2]: C 45.72, H 2.05, N 3.55, S 8.13; Found: C 45.83, H 1.99, N 3.46, S 7.95.

### 3.4. 1,4-Bis((3,7-dibromo-10H-phenothiazin-10-yl)methyl)benzene *(**4b**)*

In an oven-dried Schlenk tube with a magnetic stir bar were placed 3,7-dibromo-10*H*-phenothiazin **1a** (3.57 g, 10.0 mmol), powdered potassium hydroxide (700 mg, 12.5 mmol), and dry *N*,*N*-dimethylformamide (30.0 mL) under nitrogen. The red solution was stirred at 60 °C (oil bath) for 2 d. Then a solution of α,α′-dibromo-*para*-xylene (1.32 g, 5.00 mmol) in *N*,*N*-dimethylformamide (20.0 mL) was added dropwise to the solution. The reaction mixture was stirred at 80 °C for 16 h. After cooling to room temp the solvent was removed in vacuo. The crude product was triturated with water (2 × 200 mL) and ethanol (100 mL). After drying under vacuo compound **4b** [47] (3.18 g, 78%) was obtained as a light yellow powder, Mp 288–290 °C. *R_f_* (*n*-hexane/acetone 7:3): 0.28. ^1^H-NMR (300 MHz, dimethylsulfoxide-d_6_): *δ* 5.06 (s, 4 H), 6.67 (d, ^3^*J* = 8.7 Hz, 4 H), 7.21 (dd, ^3^*J* = 8.7 Hz, ^4^*J* = 2.3 Hz, 4 H), 7.24 (s, 4 H), 7.34 (d, ^4^*J* = 2.3 Hz, 4 H). ^13^C-NMR (500 MHz, dimethylsulfoxide-d_6_): *δ* 50.7 (CH_2_), 99.4 (C_quat_), 114.1 (CH), 117.5 (C_quat_), 124.5 (CH), 126.9 (CH), 128.6 (CH), 130.0 (C_quat_), 143.2 (C_quat_). MS (EI) *m/z*: 818 (1, [M^81^Br_3_^79^Br]^+^), 816 [M^81^Br_2_^79^Br_2_]^+^, 814 (1, [M^81^Br^79^Br_3_]^+^), 360 (9, [C_12_H_6_^81^Br_2_NS]^+^), 358 (100, [C_12_H_6_^81^Br^79^BrNS]^+^), 356 (58, [C_12_H_6_^79^Br_2_NS]^+^), 278 (74, [C_12_H_7_^81^BrNS]^+^), 276 (82, [C_12_H_7_^79^BrNS]^+^), 196 (88, [C_12_H_6_NS]^+^), 104 (32, [C_8_H_8_]^+^). IR $\tilde{\nu }$ [cm^−1^]: 2384 (vw), 2349 (vw), 2253 (vw), 1586 (w), 1485 (w), 1456 (vs), 1435 (w), 1387 (w), 1348 (m), 1296 (vw), 1258 (s), 1223 (m), 1157 (vw), 1121 (w), 1107 (m), 1082 (vw), 1045 (vw), 1007 (m), 982 (vw), 966 (vw), 930 (w), 825 (s), 795 (vs), 741 (s), 652 (m). Anal. calcd. for C_32_H_20_Br_4_N_2_S_2_ [816.3]: C 47.09, H 2.47, N 3.43, S 7.86; Found: C 46.96, H 2.60, N 3.47, S 7.62.

### 3.5. General Procedure (GP2) of Synthesis of Tetrakis(ethynyl)diphenothiazines ***7***

In an oven-dried screw-capped Schlenk vessel with a magnetic stir bar under nitrogen atmosphere were placed the tetrahalogenated dyad **4** (1.00 equiv), CuI (20.0 mol%), PdCl_2_(PPh_3_)_2_ (20.0 mol%), dry tetrahydrofuran (26.4 mL/mmol) and piperidine (13.5 mL/mmol). The reaction mixture was deaerated by a constant stream of nitrogen by a syringe for 10 min. Then, (trimethylsilyl)acetylene (**5**) (7.40 equivs) was added to the reaction mixture, which was subsequently heated at 67 °C (oil bath) under nitrogen for 21 h. After cooling to room temp, the solvents were removed under reduced pressure and the residue **6** was triturated with diethylether. The precipitated salts were separated by filtration and the solvents of the filtrate were evaporated in vacuo. The residue was purified by chromatography on silica gel. The product was dissolved in tetrahydrofuran (10.5 mL/mmol) and a 1.0 m solution of TBAF (6.00 equivs) was added. After stirring at room temp for 1 h, the solvents were removed in vacuo and the product **7** was purified by suspension in water, filtration and drying of the solid under vacuo.

#### 3.5.1. 1,4-Bis(3,7-diethynyl-10H-phenothiazin-10-yl)benzene (**7a**)

According to GP2 compound **4a** (4.21 g, 5.34 mmol), PdCl_2_(PPh_3_)_2_ (729 mg, 1.07 mmol), CuI (202 mg, 1.07 mmol), THF (140 mL), piperidine (72 mL), and (trimethylsilyl)-acetylene (5.30 mL, 39.5 mmol) were reacted to give compound **7a** (1.61 g, 53%) as an orange powder after chromatography on silica gel (hexane/dichloromethane 9:1), Mp > 400 °C (dec.). *R_f_* (hexane/dichloromethane 4:1): 0.45. ^1^H-NMR (600 MHz, tetrahydrofuran-d_8_): *δ* 3.50 (s, 4 H), 6.33 (d, ^3^*J* = 8.7 Hz, 4 H), 7.04 (dd, ^3^*J* = 8.7 Hz, ^4^*J* = 2.0 Hz, 4 H), 7.16 (d, ^4^*J* = 2.0 Hz, 4 H), 7.71 (s, 4 H). ^13^C-NMR (151 MHz, tetrahydrofuran-d_8_): *δ* 81.0 (CH), 82.5 (C_quat_), 116.4 (CH), 119.4 (C_quat_), 125.3 (CH), 129.5 (CH), 131.3 (C_quat_), 133.2 (CH), 139.6 (C_quat_), 143.0 (C_quat_). MS (MALDI-TOF) calcd. for [C_38_H_20_N_2_S_2_]^+^ *m/z*: 568.110; Found: 568.120. IR $\tilde{\nu }$ [cm^−1^]: 3287 (m), 2104 (m), 1599 (m), 1582 (m), 1503 (w), 1495 (w), 1468 (vw), 1387 (s), 1312 (vw), 1256 (vw), 1190 (w), 1152 (w), 1096 (w), 1077 (w), 1065 (vw), 1045 (vw), 1020 (m), 937 (m), 880 (s), 866 (w), 806 (vw), 779 (vw), 745 (w), 729 (w), 710 (w), 667 (s), 648 (vw). Anal. calcd. for C_38_H_20_N_2_S_2_ (568.7): C 80.25, H 3.54, N 4.93, S 11.27; Found: C 80.01, H 3.60, N 4.71, S 10.99.

#### 3.5.2. 1,4-Bis((3,7-diethynyl-10H-phenothiazin-10-yl)methyl)benzene (**7b**)

According to GP2 compound **4b** (2.86 g, 3.50 mmol), PdCl_2_(PPh_3_)_2_ (491 mg, 700 μmol), CuI (133 mg, 700 μmol), THF (92 mL), piperidine (47 mL), and (trimethylsilyl)acetylene (3.69 mL, 25.9 mmol) were reacted to give compound **7b** (1.13 g, 54%) as an orange powder after chromatography on silica gel (hexane/dichloromethane 9:1), Mp >400 °C (dec.). *R_f_* (hexane/dichloromethane 4:1): 0.29. ^1^H-NMR (600 MHz, tetrahydrofuran-d_8_): *δ* 3.47 (s, 4 H), 5.11 (s, 4 H), 6.66 (d, ^3^*J* = 8.5 Hz, 4 H), 7.08 (dd, ^3^*J* = 8.5 Hz, ^4^*J* = 1.8 Hz, 4 H), 7.16 (d, ^4^*J* = 1.8 Hz, 4 H), 7.27 (s, 4 H). ^13^C-NMR (151 MHz, tetrahydrofuran-d_8_): *δ* 79.0 (CH), 83.5 (CH), 116.6 (CH), 118.1 (C_quat_), 124.0 (C_quat_), 128.1 (CH), 130.9 (CH), 132.2 (CH), 136.5 (C_quat_), 145.5 (C_quat_). MS (MALDI-TOF) calcd. for [C_40_H_24_N_2_S_2_]^+^ *m/z*: 596.770; Found: 597.160. IR $\tilde{\nu }$ [cm^−1^]: 3298 (w), 3055 (vw), 2108 (vw), 1599 (w), 1464 (vs), 1456 (s), 1400 (m), 1354 (m), 1339 (w), 1300 (w), 1256 (m), 1223 (s), 1163 (w), 1123 (w), 1007 (w), 1065 (w), 1011 (w), 893 (m), 881 (m), 816 (s), 745 (w), 704 (w), 633 (s), 604 (m). Anal. calcd. for C_40_H_24_N_2_S_2_ [596.8] C 80.51, H 4.05, N 4.69, S 10.74; Found: C 79.65, H 4.25, N 4.37, S 10.53.

### 3.6. General Procedure (GP3) of the CuAAC Synthesis of Tetrakis[(triethoxysilylpropyl)triazole] Substituted Diphenothiazines ***8***

In an oven-dried screw-capped Schlenk vessel with a magnetic stir bar under nitrogen atmosphere were placed the tetrakis(alkynylated) dyad **7** (1.00 equiv), CuI (10.0 mol% per alkynyl unit) and dry DMF (22.4 mL/mmol). After addition of (3-azidopropyl)tri-ethoxysilane (**2a**) (1.00 equiv per alkynyl unit), the turbid reaction mixture was stirred at room temp for 16 h, while the reaction mixture turned red. The solvent was removed under reduced pressure and the residue was purified by chromatography on silica gel.

#### 3.6.1. 1,4-Bis(3,7-bis(1-(3-(triethoxysilyl)propyl)-1*H*-1,2,3-triazol-4-yl)-10*H*-phenothiazin-10-yl)benzol (**8a**)

According to GP 3 compound **7a** (260 mg, 457 μmol), CuI (34.8 mg, 183 μmol), CuI (34.8 mg, 183 μmol), (3-azidopropyl)triethoxysilane (**2a**) (588 mg, 2.38 mmol), and DMF (27 mL) were reacted to give compound **8a** (235 mg, 33%) as a yellow crystalline solid after chromatography on silica gel (hexane/acetone 6:4), Mp > 350 °C (dec.). *R_f_* (*n*-hexane/acetone 6:4): 0.15. ^1^H-NMR (300 MHz, tetrahdrofuran-d_8_): *δ* 0.62 (m_c_, 8 H), 1.19 (t, ^3^*J* = 7.0 Hz, 36 H), 2.02 (m_c_, 8 H), 3.80 (q, ^3^*J* = 7.0 Hz, 24 H), 4.36 (t, ^3^*J* = 6.8 Hz, 8 H), 6.57 (d, ^3^*J* = 8.5 Hz, 4 H), 7.54 (dd, ^3^*J* = 8.5 Hz, ^4^*J* = 2.0 Hz, 4 H), 7.63 (d, ^4^*J* = 2.0 Hz, 4 H), 7.67 (s, 4 H), 8.09 (s, 4 H). ^13^C-NMR (75 MHz, tetrahdrofuran-d_8_): *δ* 14.1 (CH_2_), 18.7 (CH_3_), 20.8 (CH_2_), 53.1 (CH_2_), 59.1 (CH_2_), 118.7 (CH), 120.2 (CH), 123.4 (C_quat_), 124.6 (CH), 125.0 (CH), 128.1 (C_quat_), 132.1 (CH), 141.9 (C_quat_), 143.8 (C_quat_), 146.8 (C_quat_). MS (MALDI-TOF) calcd. for [C_74_H_104_N_14_O_12_S_2_Si_4_]^+^ *m/z*: 1558.190; Found: 1556.650. IR $\tilde{\nu }$ [cm^−1^]: 2953 (m), 2922 (m), 2853 (m), 1653 (vw), 1601 (vw), 1458 (w), 1437 (w), 1364 (w), 1310 (w), 1271 (m), 1248 (vs), 1233 (s), 1167 (m), 1119 (w), 1086 (m), 1034 (s), 945 (w), 930 (w), 912 (vw), 874 (w), 858 (vw), 800 (m), 768 (m), 743 (vw), 723 (vw), 700 (vw), 644 (s), 637 (s), 621 (m).

#### 3.6.2. 1,4-Bis((3,7-bis(1-(3-(triethoxysilyl)propyl)-1*H*-1,2,3-triazol-4-yl)-10*H*-phenothiazin-10-yl)methyl)benzene (**8b**)

According to GP 3 compound **7b** (148 mg, 248 μmol), CuI (18.9 mg, 99.2 μmol), CuI (34.8 mg, 183 μmol), (3-azidopropyl)triethoxysilane (**2a**) (319 mg, 1.29 mmol), and DMF (15 mL) were reacted to give compound **8b** (138 mg, 35%) as yellow crystals after chromatography on silica gel (hexane/acetone 6:4), Mp >320 °C (dec.). *R_f_* (hexane/acetone 6:4): 0.17. ^1^H-NMR (500 MHz, tetrahydrofuran-d_8_): *δ* 0.65 (m_c_, 8 H), 1.18 (t, ^3^*J* = 7.0 Hz, 36 H), 2.73 (m_c_, 8 H), 3.82 (q, ^3^*J* = 7.0 Hz, 24 H), 4.44 (t, ^3^*J* = 7.1 Hz, 8 H), 4.46 (s, 4 H), 6.61 (d, ^3^*J* = 8.6 Hz, 4 H), 7.56 (dd, ^3^*J* = 8.6 Hz, ^4^*J* = 2.0 Hz, 4 H), 7.67 (d, ^4^*J* = 2.0 Hz, 4 H), 7.77 (s, 4 H), 8.29 (s, 4 H). ^13^C-NMR (125 MHz, tetrahdrofuran-d_8_): *δ* 8.2 (CH_2_), 18.7 (CH_3_), 25.1 (CH_2_),52.3 (CH_2_), 53.1 (CH_2_), 59.0 (CH_2_), 118.6 (CH), 120.9 (CH), 122.6 (C_quat_), 124.6 (CH), 125.3 (CH), 127.9 (C_quat_), 132.8 (CH), 141.7 (C_quat_), 143.9 (C_quat_), 146.7 (C_quat_). MS (MALDI-TOF) calcd. for [C_76_H_108_N_14_O_12_S_2_Si_4_]^+^ *m/z*: 1586.250; Found: 1586.000. IR $\tilde{\nu }$ [cm^−1^]: 2818 (vw), 2365 (w), 1597 (vw), 1456 (vs), 1414 (w), 1331 (m), 1258 (m), 1242 (m), 1198 (w), 1155 (w), 1140 (m), 1107 (w), 1038 (w), 1009 (vw), 961 (vw), 910 (vw), 872 (w), 862 (w), 806 (s), 745 (s), 725 (m), 706 (m), 650 (w).

### 3.7. Synthesis of Hybrid Materials **3b–d@MCM-41**

#### 3.7.1. Synthesis of Hybrid Material **3b@MCM-41**

An aqueous solution of cetyltrimethylammonium bromide (CTAB) was mixed with ethylamine under stirring. Then, a solution of precursor **3b** in methanol and tetraethylorthosilicate (TEOS) was added dropwise. The composition of the mixture of TEOS:**3b**:CTAB:EtNH_2_:methanol:H_2_O in molar ratio was 1.0:0.085:0.14:2.4:2.0:100. The reaction mixture was stirred at room temp for 24 h to obtain a homogenous mixture of the organosilane with TEOS. Then the mixture was stirred at 100 °C for 24 h. The product was collected by filtration, washed thoroughly with deionized water to pH-Neutrality. After drying at 50 °C under vacuo a powdery material was obtained. For extraction of the surfactant (CTAB) 1.0 g of the powder was suspended twice in ethanol (80 mL) and aqueous HCl (36%, 1.0 mL) and heated to reflux for 8 h. After cooling to room temp, the solid was filtered, washed with ethanol and dichloromethane and finally dried at 50 °C under vacuo. The material **3b@MCM-41** was obtained as a yellow powder. Anal. calcd for **3b@MCM-41**: C 20.63, H 3.34, N, 3.94; which corresponds to a loading of 0.70 mmol/g.

#### 3.7.2. Synthesis of Hybrid Material **3c-1@MCM-41**

An aqueous solution of cetyltrimethylammonium bromide (CTAB) was mixed with ethylamine under stirring. Then a solution of precursor **3c** in methanol/THF and tetraethylorthosilicate (TEOS) was added dropwise. The composition of the mixture of TEOS:**3c**:CTAB:EtNH_2_:methanol:THF:H_2_O in molar ratio was 1.0:0.03:0.14:2.4:2.0:2.0:100. The reaction mixture was stirred at room temp for 24 h to obtain a homogenous mixture of the organosilane with TEOS. Then the mixture was stirred at 100 °C for 24 h. The product was collected by filtration, washed thoroughly with deionized water to pH-Neutrality. After drying at 50 °C under vacuo a powdery material was obtained. For extraction of the surfactant (CTAB), 1.0 g of the powder was suspended twice in ethanol (80 mL) and aqueous HCl (36%, 1.0 mL) and heated to reflux temp for 8 h. After cooling to room temp, the solid was filtered, washed with ethanol and dichloromethane and finally dried at 50 °C under vacuo. The material **3c-1@MCM-41** was obtained as a yellow powder. Anal. calcd for **3c-1@MCM-41**: C 19.92, H 2.92, N, 1.93; which corresponds to a loading of 0.28 mmol/g.

#### 3.7.3. Synthesis of Hybrid Material **3c-2@MCM-41**

An aqueous solution of cetyltrimethylammonium bromide (CTAB) was mixed with ethylamine under stirring. Then, a solution of precursor **3c** in methanol/THF and tetraethylorthosilicate (TEOS) was added dropwise. The composition of mixture TEOS:**3c**:CTAB:EtNH_2_:methanol:THF:H_2_O in molar ratio was 1.0:0.055:0.14:2.4:2.0:2.0:100. The reaction mixture was stirred at room temp for 24 h to obtain a homogenous mixture of the organosilane with TEOS. Then the mixture was stirred at 100 °C for 24 h. The product was collected by filtration, washed thoroughly with deionized water to pH-neutrality. After drying at 50 °C under vacuo a powderous material was obtained. For extraction of the surfactant (CTAB) 1.0 g of the powder was twice suspended in ethanol (80 mL) and aqueous 36% HCl (1.0 mL) and heated to reflux temp for 8 h. After cooling to room temp the solid was filtered, washed with ethanol and dichloromethane and finally dried at 50 °C under vacuo. The material **3c-2@MCM-41** was obtained as a yellow powder. Anal. calcd for **3c-2@MCM-41**: C 27.79, H 2.97, N, 3.04; which corresponds to a loading of 0.43 mmol/g.

#### 3.7.4. Synthesis of Hybrid Material **3d@MCM-41**

An aqueous solution of cetyltrimethylammonium bromide (CTAB) was mixed with ethylamine under stirring. Then, a solution of precursor **3d** in methanol/THF and tetraethylorthosilicate (TEOS) was added dropwise. The composition of the mixture of TEOS:**3d**:CTAB:EtNH_2_:methanol:THF:H_2_O in molar ratio was 1.0:0.04:0.14:2.4:2.0:2.0:100. The reaction mixture was stirred at room temp for 24 h to obtain a homogenous mixture of the organosilane with TEOS. Then the mixture was stirred at 100 °C for 24 h. The pro-duct was collected by filtration, washed thoroughly with deionized water to pH-Neutrality. After drying at 50 °C under vacuo a powdery material was obtained. For extraction of the surfactant (CTAB), 1.0 g of the powder was suspended twice in ethanol (80 mL) and aqueous HCl (36%, 1.0 mL) and heated to reflux temp for 8 h. After cooling to room temp, the solid was filtered, washed with ethanol and dichloromethane and finally dried at 50 °C under vacuo. The material **3d@MCM-41** was obtained as a yellow powder. Anal. calcd for **3d@MCM-41**: C 12.67, H 2.82, N, 2.78; which corresponds to a loading of 0.28 mmol/g.

## 4. Conclusions

In summary, we have successfully employed the CuAAC, a copper catalyzed variation of (3+2)-cycloadditions, for synthesizing triazole conjugated phenothiazines bearing propyl trialkoxylsilyl moieties as precursors for the synthesis of mesoporous silica hybrid materials by the in-situ co-condensation strategy. The precursor materials are all intensively luminescent by triazole substitution and reversible redox systems according to cyclic voltammetry. These favorable electronic properties can be directly included in the mesoporous hybrid materials as well. Exemplarily, four different materials were synthesized and extensively characterized for XRD, nitrogen absorption-desorption isotherms, TEM, solid state CP-MAS NMR and IR spectroscopy to gain an insight into mesoporous structuring and molecular ligation in the silica framework. The mesoporous periodicity of the obtained materials largely depends on the molecular dimensions of the employed precursors in the templated in-situ condensation synthesis. The electronic properties of the hybrid materials are affected by the molecular properties of the constituents, however, more dense packing and embedding leads to further cooperativity, e.g., intense fluorescence. Also, the stability of chemically oxidized materials displaying the formation of radical cations sets the stage for devising chemically and electrochemically reversible electrophores embedded in stabilizing silica frameworks. Further studies will be directed to develop periodically mesoporous materials with switchable charge transport properties.

## Figures and Tables

**Table 1:** Selected electronic properties (cyclic voltammetric oxidation potentials, absorption and emission data by UV/Vis and fluorescence) of the triazolyl conjugated (oligo)phenothiazines **3** and **8**.

Compound	Oxidation Potentials ^1^		Absorption ^2^	Emission ^3^	Stokes Shift ^4^
	*E_1/2_^0/+1^*	*E_1/2_^+1/+2^*	*λ*_max,abs_	*λ*_max,abs_	$\Delta \tilde{\nu }$
	[mV]	[mV]	[nm] (*ε* [m^−1^cm^−1^])	[nm]	[cm^−1^]
**3a**	750	-	266 (49200), **319** (9950)	**446**, 469sh	8900
**3b**	720	-	268 (55400), **318** (14250)	**450**	9200
**3c**	650	790	278 (43200), 328 (14600), **367** (12300)	**470**, 505sh	6000
**3d**	730	-	276 (37800), **334** (7200)	459	8100
**3e**	700	-	276 (54500), **335** (10100)	**460**, 484sh	8100
**3f**	650	820	278 (50700), **336** (16100)	**469**, 506sh	5700
**8a** ^5^	700	880	279 (183000), **343** (20000)	**455**, 479	7200
**8b** ^5^	800	-	274 (83000), **335** (10000)	**456**, 485	7900

^1^ Recorded in dichloromethane at *T* = 298 K, *ν* = 100 mVs^−1^, electrolyte: [Bu_4_N][PF_6_], Pt working electrode, Pt counter electrode, Ag/AgCl reference electrode, referenced to *E_1/2_^0/+1^*(Fc/Fc^+^) = 450 mV. ^2^ Recorded in dichloromethane at *T* = 298 K, *c* = 10^−3^ m. ^3^ Recorded in dichloromethane at *T* = 298 K, *c* = 10^−6^ m (excitation at the longest wavelength absorption band). ^4^ $\Delta \tilde{\nu }$ = 1/*λ*_max,abs_−1/*λ*_max,em_ [cm^−1^] (the corresponding absorption and emission bands for determining the Stokes shifts are high-lighted in **bold face**). ^5^ Cyclic voltammograms referenced to *E_1/2_^0/+1^*(Me_10_Fc/Me_10_Fc^+^) = −95 mV.

**Table 2:** Textural parameters of **MCM-41** [30] and **3b**–**d@MCM-41** derived from powder X-ray diffraction and nitrogen adsorption-desorption analyses and the phenothiazine loading in the corresponding hybrid materials.

Sample	Content of Phenothiazine ^1^		*d*_100_^2^	*a*_0_^3^	*S*_BET_^4^	*V*_p_^5^	*D*_p_ ^6^	*w*_t_ ^7^
	[mmol g^–1^]	[wt%]	[nm]	[nm]	[m^2^g^–^^1^]	[cm^3^g^–1^]	[nm]	[nm]
**MCM-41**	−	−	4.30	4.96	1123	0.98	2.83	2.13
**3b@MCM-41**	0.70	30.4	4.20	4.85	536	0.36	2.36	2.49
**3c-1@MCM-41**	0.28	18.3	4.44	5.13	1086	0.96	2.82	2.31
**3c-2@MCM-41**	0.43	28.1	4.50	5.20	543	0.48	2.77	2.43
**3d@MCM-41**	0.28	14.9	4.48	5.17	539	0.41	2.41	2.76

^1^ Calculated from the CHN combustion analysis according to the content of nitrogen. ^2^ *d*_100_ is the d(100) spacing. ^3^ *a*_0_ is the cell parameter (*a*_0_ = 2d_100_/√3). ^4^ *S*_BET_ is the BET surface area. ^5^ V_p_ is the pore volume. ^6^ *D*_p_ is the pore diameter; ^7^ *w*_t_ is the wall thickness *a*_0_-*D*_p_.

**Table 3:** Selected optical properties (absorption and emission data by UV/Vis and fluorescence) of the triazolyl conjugated (oligo)phenothiazines **3b**–**d** ^1^ and hybrid materials **3b**–**d@MCM-41** ^2^.

Compound	Absorption	Emission	Stokes Shift ^3^
	*λ*_max,abs_	*λ*_max,abs_	Δ$\tilde{\nu }$
	[nm]	[nm]	[cm^−1^]
**3b**	268, **318**	**450**	9200
**3b@MCM-41**	260, **320**	**451**	9100
**3c**	278, 328, **367**	**470**, 505sh	6000
**3c-1@MCM-41**	276, 330, **370**	**484**, 509sh	6400
**3c-2@MCM-41**	275, 327, **376**	**500**	6700
**3d**	276, **334**	**459**	8100
**3d@MCM-41**	270, 327, **360**	390, **445**	5300

^1^ Recorded in dichloromethane at *T* = 298 K (emission spectra were recorded by excitation at the longest wavelength absorption band). ^2^ UV/Vis spectra of diffuse reflectance. ^3^ $\Delta \tilde{\nu }$ = 1/*λ*_max,abs_ − 1/*λ*_max,em_ [cm^−1^] (the corresponding absorption and emission bands for determining the Stokes shifts are highlighted in **bold face**).

**Table 4:** Experimental details of the CuAAC synthesis of precursors **3**.

Entry	Alkyne 1	Azide 2	CuI	DMF	Product 3 Yield
	[mg] ([μmol])	[mg] ([μmol])	[mg] ([μmol])	[mL]	[mg] (%)
1	100 (422) of **1a**	105 (422) of **2a**	8.0 (42)	3 mL	193 (95) of **3a**
2	129 (422) of **1b**	105 (422) of **2a**	8.0 (42)	3 mL	228 (97) of **3b**
3	735 (1250) of **1c**	256 (1250) of **2b**	24.0 (125)	7 mL	970 (98) of **3c**
4	55 (211) of **1d**	105 (422) of **2a**	4.0 (42)	3 mL	140 (88) of **3d**
5	270 (815) of **1e**	402 (1630) of **2a**	16.0 (82)	3 mL	625 (93) of **3e**
6	59 (96) of **1f**	41 (200) of **2b**	1.9 (10)	3 mL	90 (92) of **3f**

## Data Availability

All data of this work are included in the manuscript and the Supplementary Materials.

## Supplementary Materials

The following are available online, Supporting Information: ^1^H- and ^13^C-NMR spectra of compounds **3** and **8**, textural parameters and powder XRD measurements, N_2_ adsorption/desorption isotherms, electron microscopy (TEM, SEM), FT-IR spectra, ^13^C CP MAS NMR spectra, ^29^Si CP MAS NMR spectra, absorption and emission spectra of hybrid materials **3b**–**d@MCM-41** and absorption spectra of oxidized and protonated hybrid materials.

## Sample Availability

Not available.
