# Supplementary material for: Triazolyl Conjugated (Oligo)Phenothiazines Building Blocks for Hybrid Materials—Synthesis and Electronic Properties
Source: Molecules. 2021 May 15;26(10):2950. doi: 10.3390/molecules26102950 (PMC8156644; doi:10.3390/molecules26102950)
Supplement: Supplementary file 1 [file molecules-26-02950-s001.zip › molecules-1224014-SI.pdf]

Supporting Information

Triazolyl Conjugated (Oligo)Phenothiazines Building Blocks for Hybrid Materials – Synthesis and Electronic Properties

Hilla Khelwati [1], Adam W. Franz [1], Zhou Zhou [2],
Werner R. Thiel [2,*] and Thomas J. J. Müller [1,*]

[1] Institut für Organische Chemie und Makromolekulare Chemie, Heinrich-Heine-Universität Düsseldorf, Universitätsstrasse 1, D-40225 Düsseldorf, Germany; ThomasJJ.Mueller@uni-duesseldorf.de

[2] Fachbereich Chemie, Technische Universität Kaiserslautern, Erwin-Schrödinger-Str. 54, D-67663 Kaiserslautern, Germany; thiel@chemie.uni-kl.de

Table of Contents

1. ^{1}H and ^{13}C NMR Spectra of the Precursors **3** and **8** ... 2
2. Textural parameters of **3b-d@MCM-41** ... 10
2.1. Powder XRD measurements and mesostructure of the hybrid materials **3b-d@MCM-41** ... 10
2.1.1. Hybrid material **3b@MCM-41** ... 10
2.1.2. Hybrid materials **3c@MCM-41** ... 11
2.1.3. Hybrid material **3d@MCM-41** ... 12
2.1.4. N_2 adsorption/desorption isotherms and pore structure of the hybrid materials **3b-d@MCM-41** ... 13
2.2. Electron microscopy of hybrid materials **3b-d@MCM-41** ... 16
2.2.1. TEM of **3b@MCM-41** ... 16
2.2.2. TEM of **3c@MCM-41** ... 17
2.2.3. TEM of **3d@MCM-41** ... 18
2.3. Spectroscopic characterization of hybrid materials **3b-d@MCM-41** ... 20
2.3.1. FT-IR spectra of hybrid materials **3b-d@MCM-41** ... 20
2.3.2. ^{13}C CP MAS NMR spectra of hybrid materials **3b-d@MCM-41** ... 22
2.3.3. ^{29}Si CP MAS NMR spectra of hybrid materials **3b-d@MCM-41** ... 24
2.4. Optical Properties of Hybrid Materials **3b-d@MCM-41** and Selected Oxidized Materials ... 26
3. References ... 29

1. ^1H and ^{13}C NMR Spectra of the Precursors 3 and 8

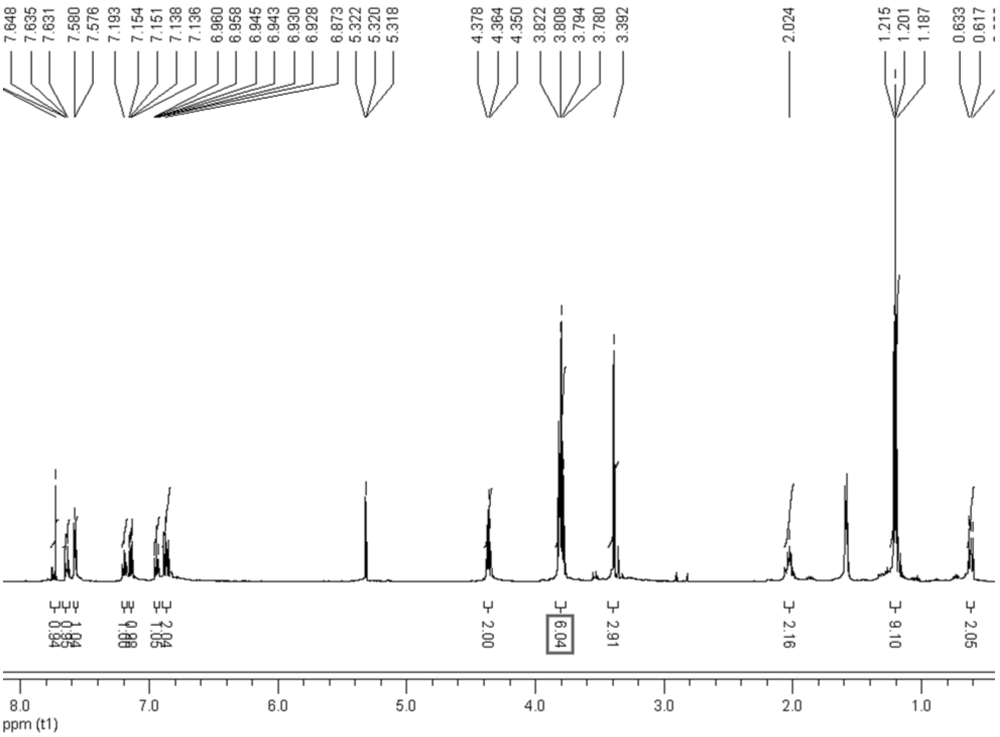

Figure S1. ^1H NMR spectrum of compound **3a** (500 MHz, CD_2Cl_2, RT).

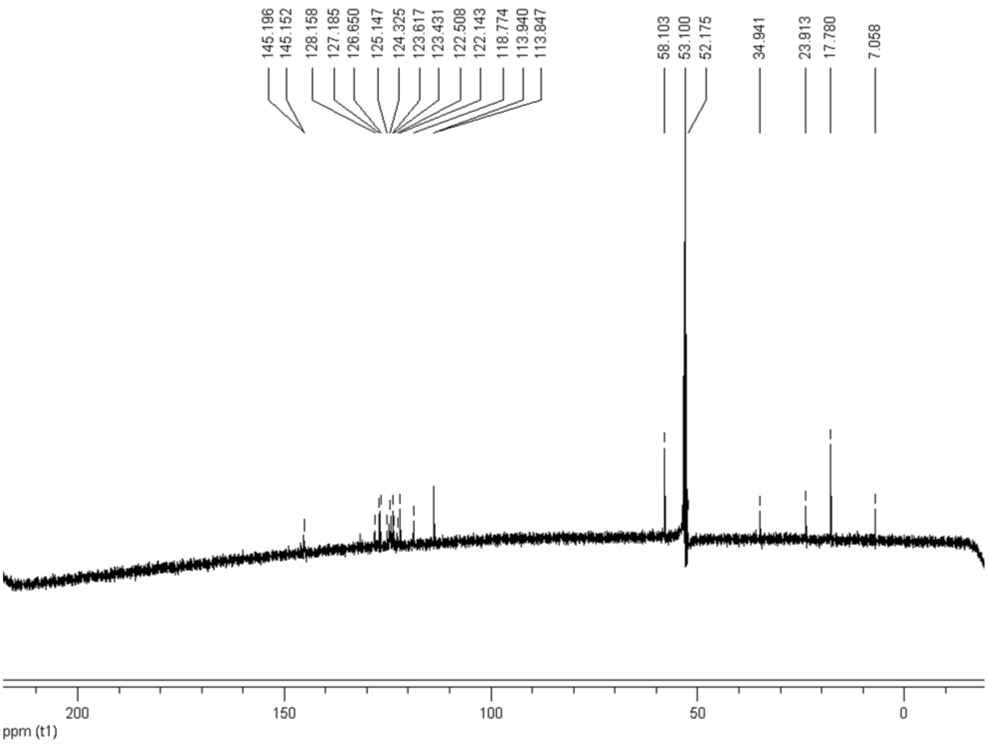

Figure S2. ^{13}C NMR spectrum of compound **3a** (125 MHz, CD_2Cl_2, RT).

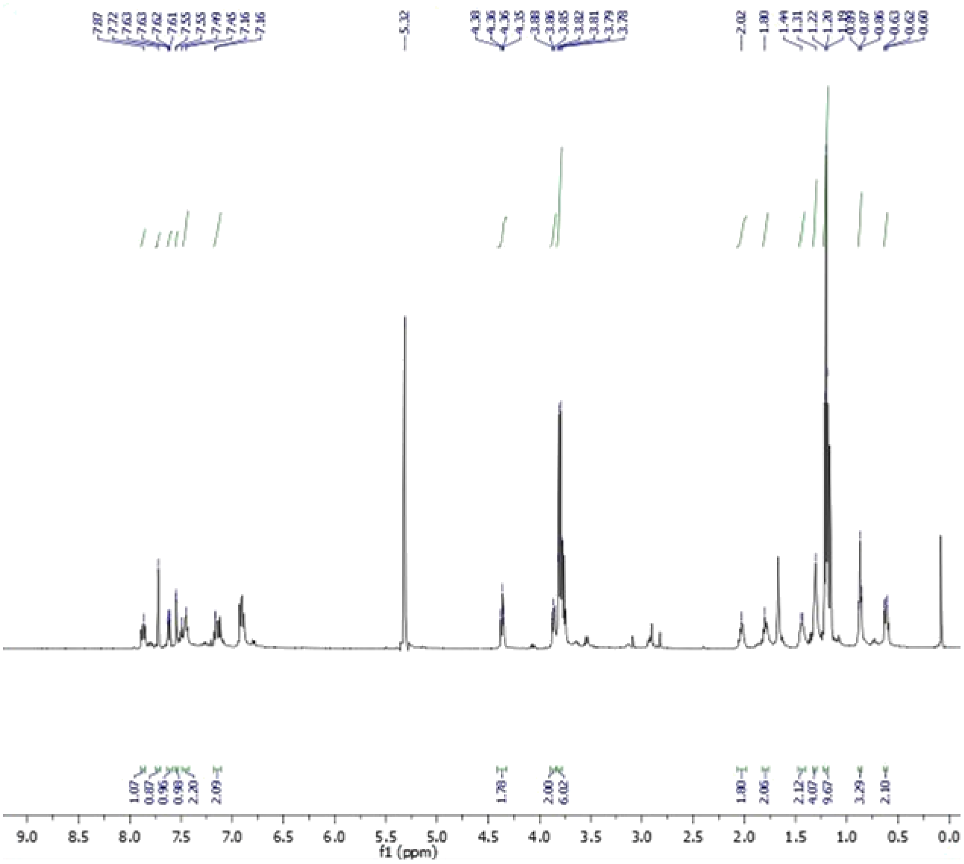

Figure S3. ^{1}H NMR spectrum of compound **3b** (500 MHz, CD_2Cl_2, RT).

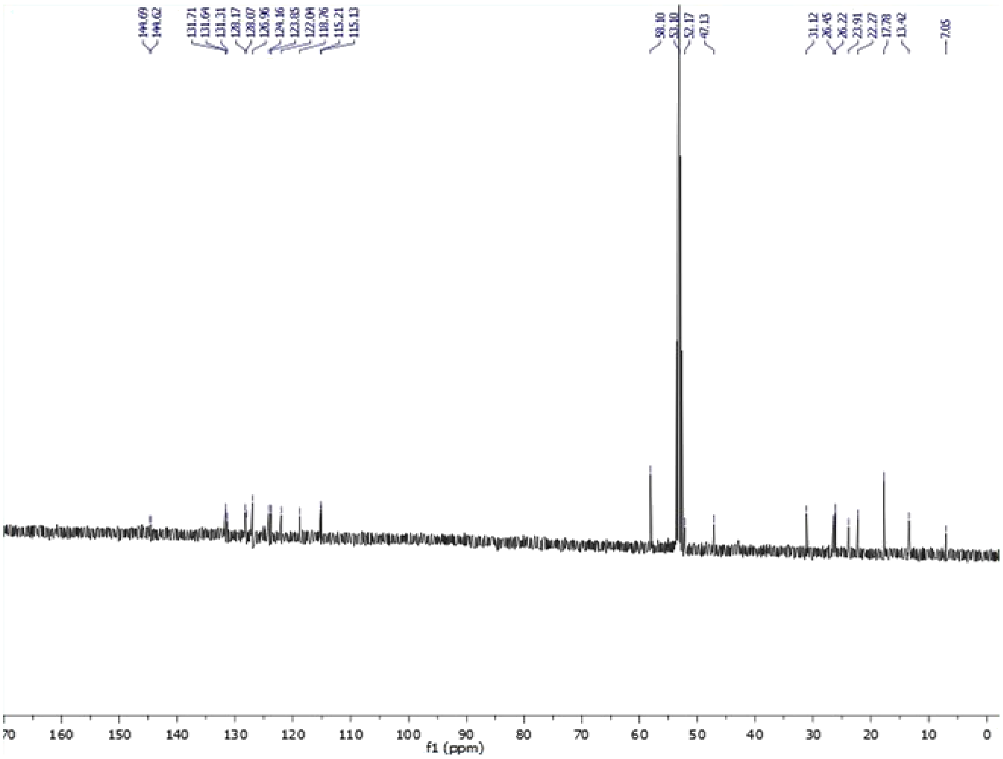

Figure S4. ^{13}C NMR spectrum of compound **3b** (125 MHz, CD_2Cl_2, RT).

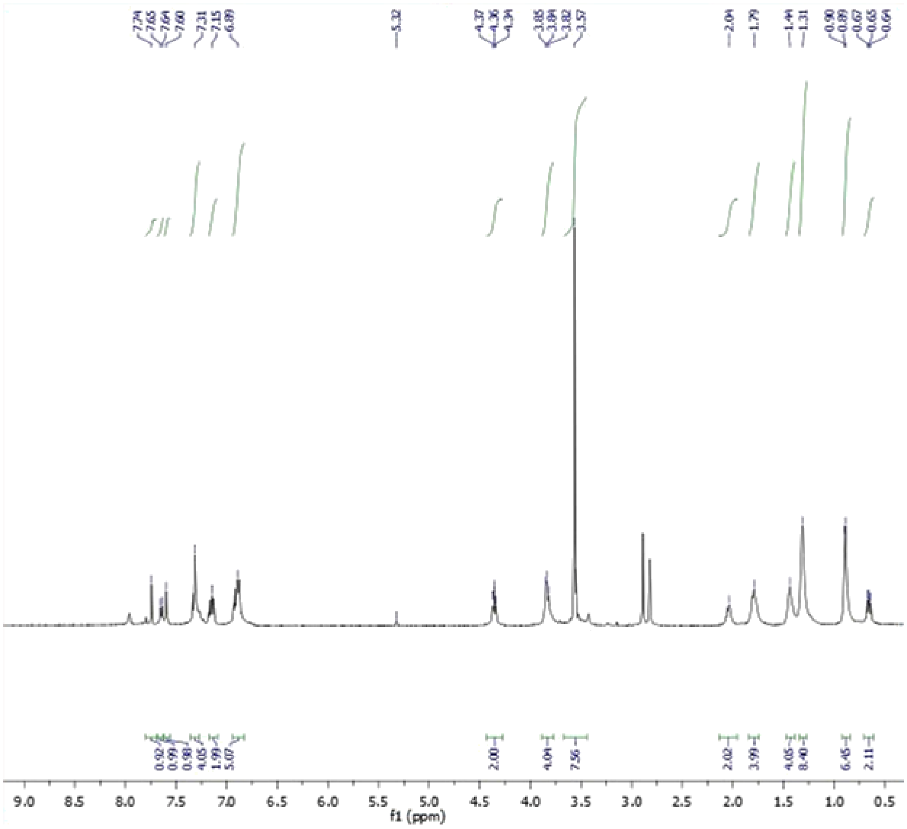

Figure S5. ^{1}H NMR spectrum of compound **3c** (500 MHz, CD_2Cl_2, RT).

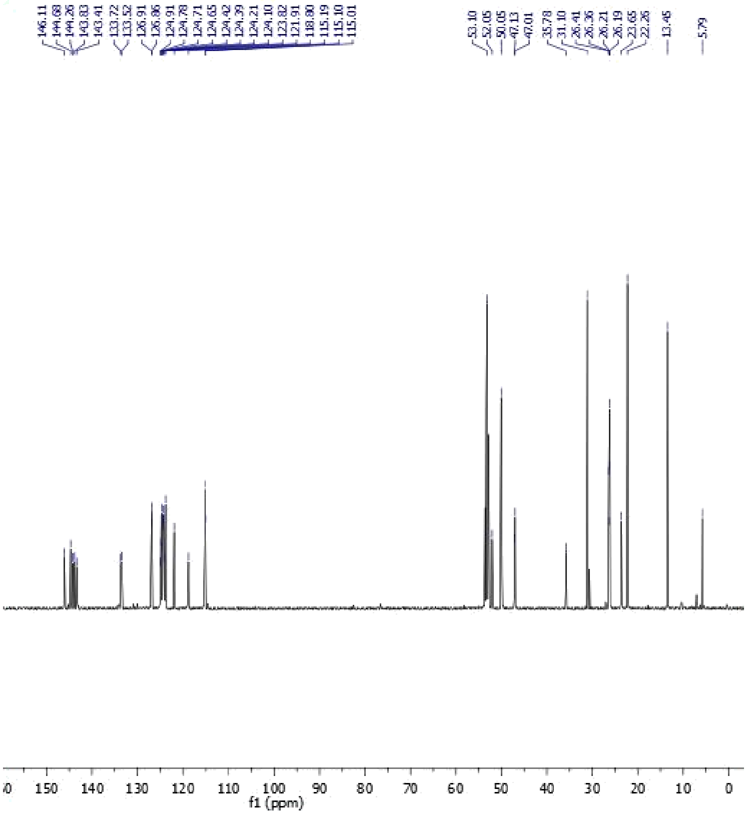

Figure S6. ^{13}C NMR spectrum of compound **3c** (125 MHz, CD_2Cl_2, RT).

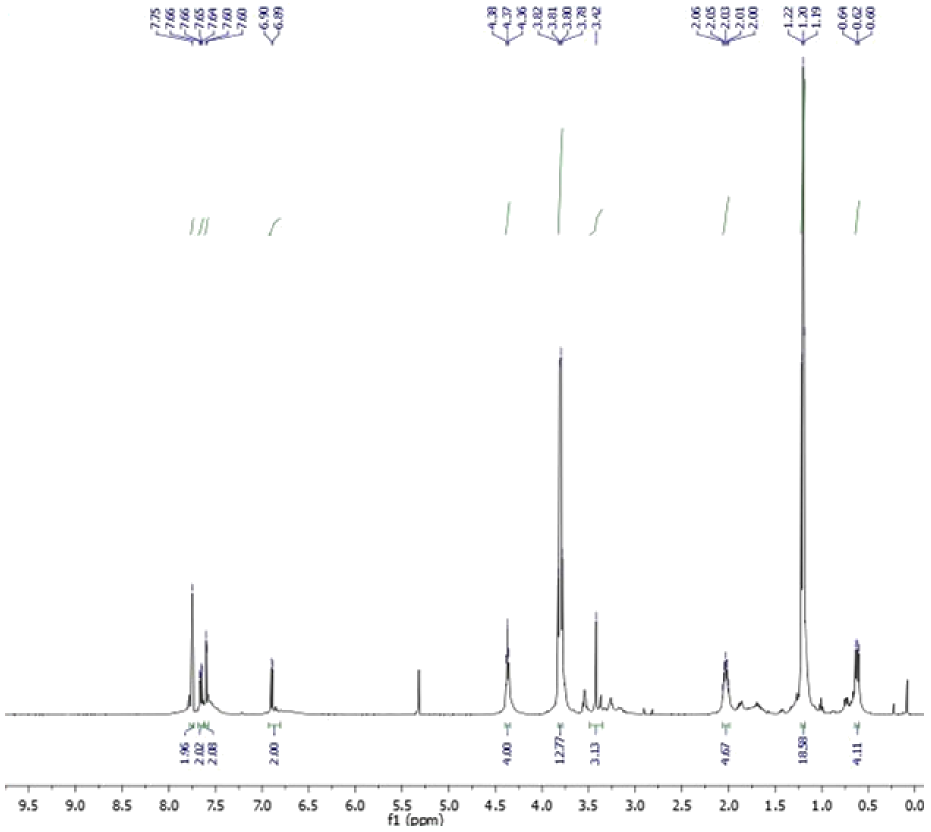

Figure S7. ^{1}H NMR spectrum of compound **3d** (500 MHz, CD_2Cl_2, RT).

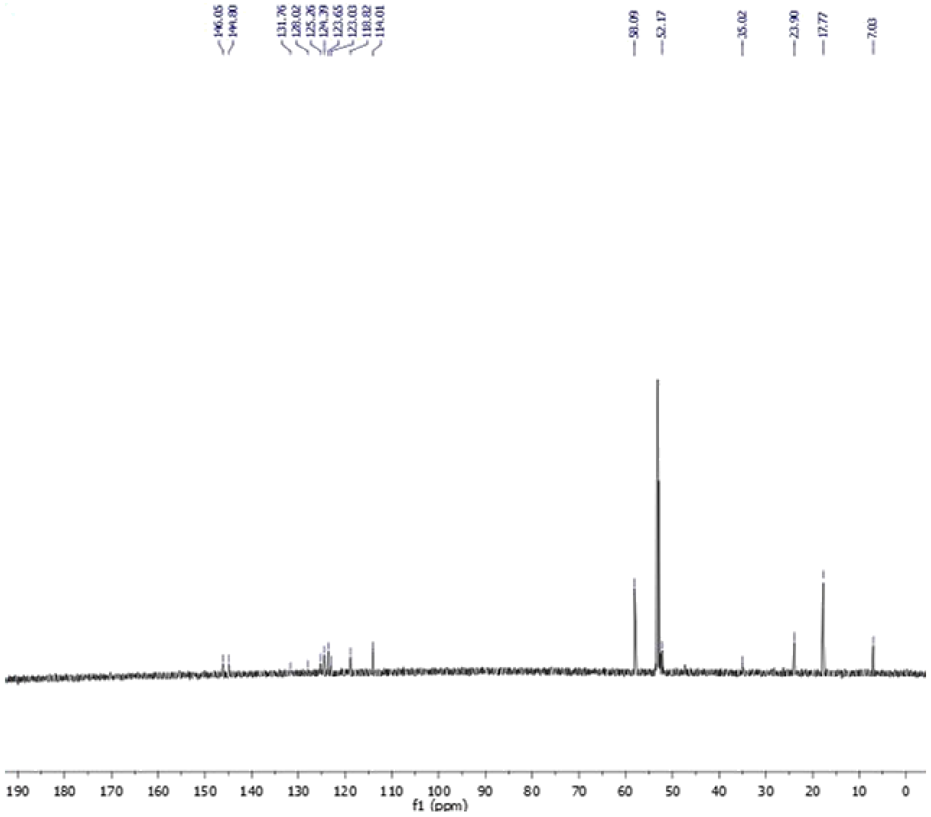

Figure S8. ^{13}C NMR spectrum of compound **3d** (125 MHz, CD_2Cl_2, RT).

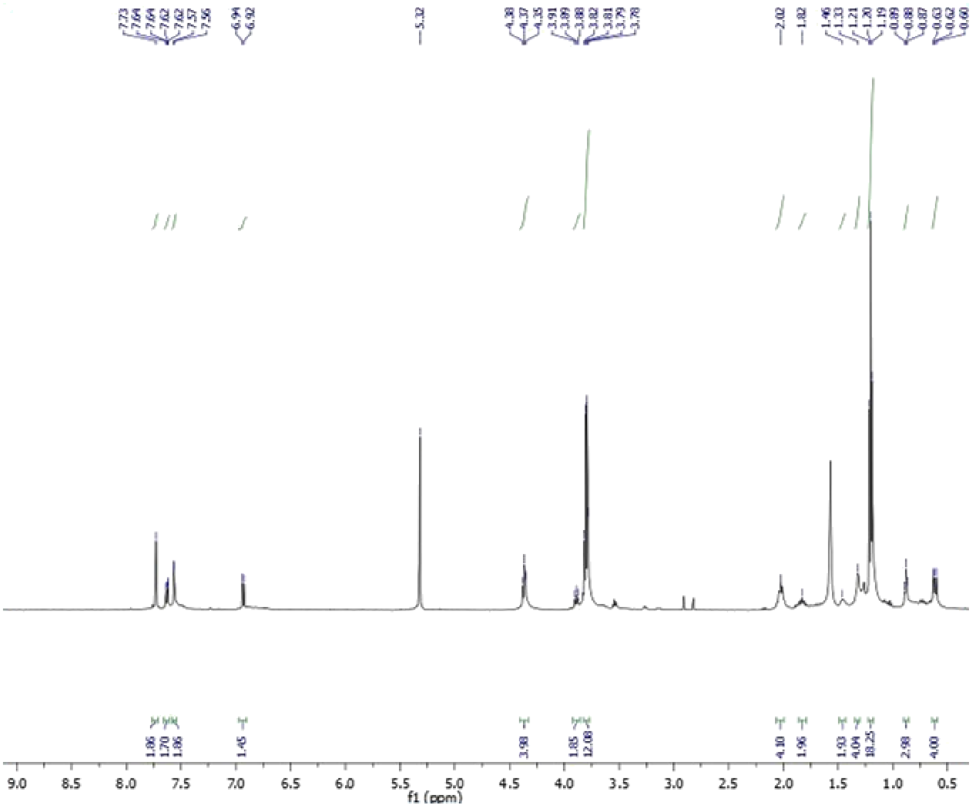

Figure S9. ^{1}H NMR spectrum of compound **3e** (500 MHz, CD_2Cl_2, RT).

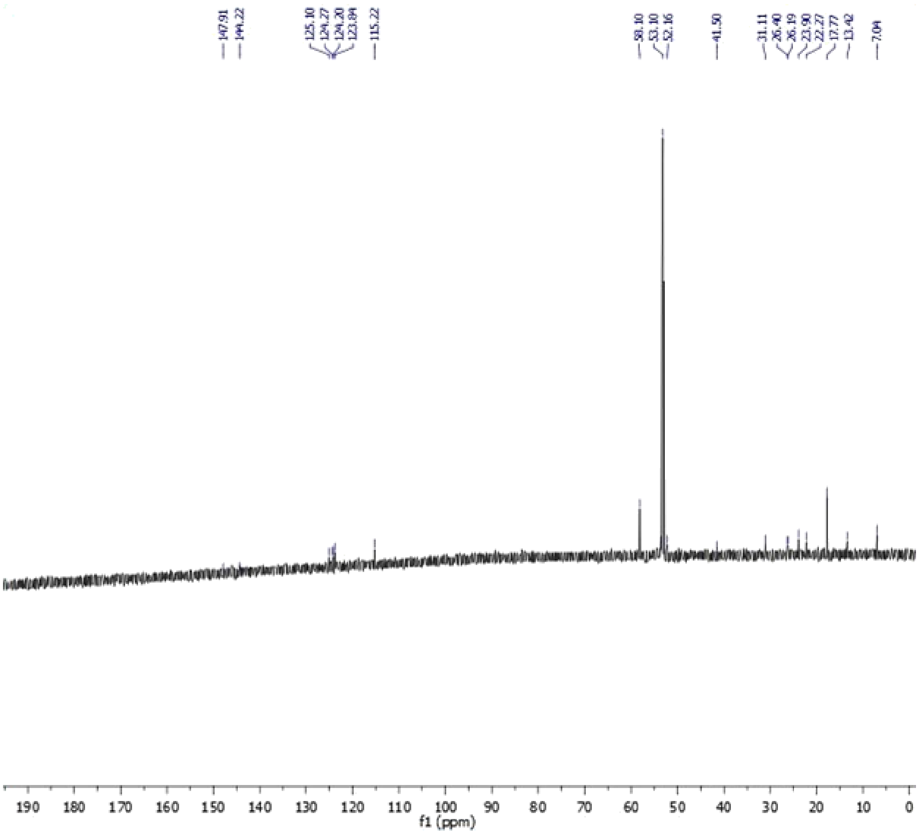

Figure S10. ^{13}C NMR spectrum of compound **3e** (125 MHz, CD_2Cl_2, RT).

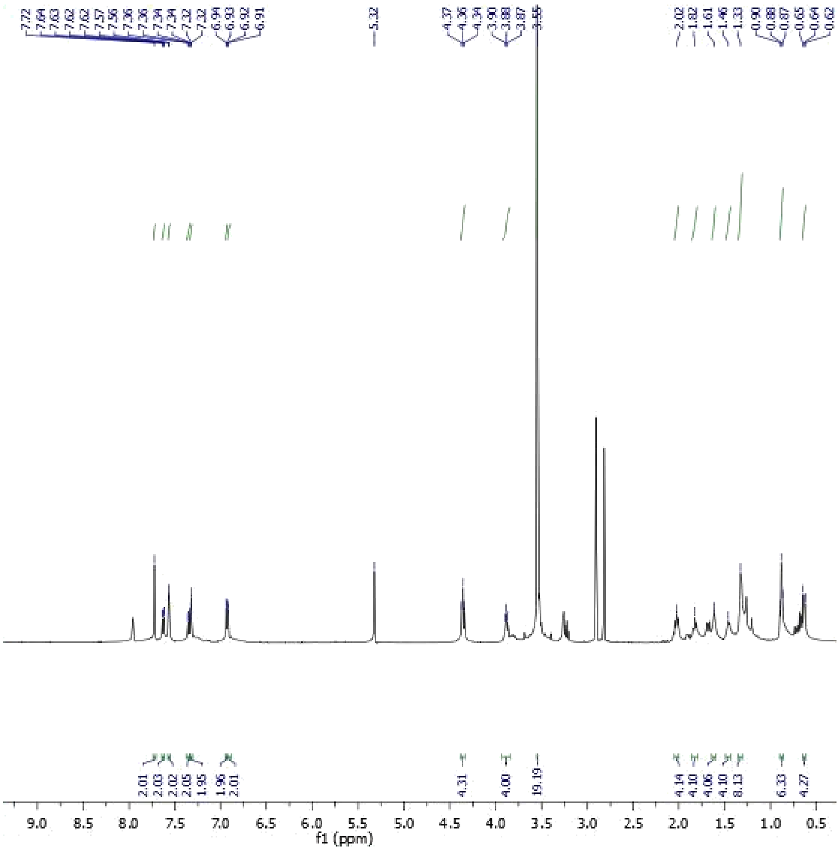

Figure S11. ^{1}H NMR spectrum of compound **3f** (500 MHz, CD_2Cl_2, RT).

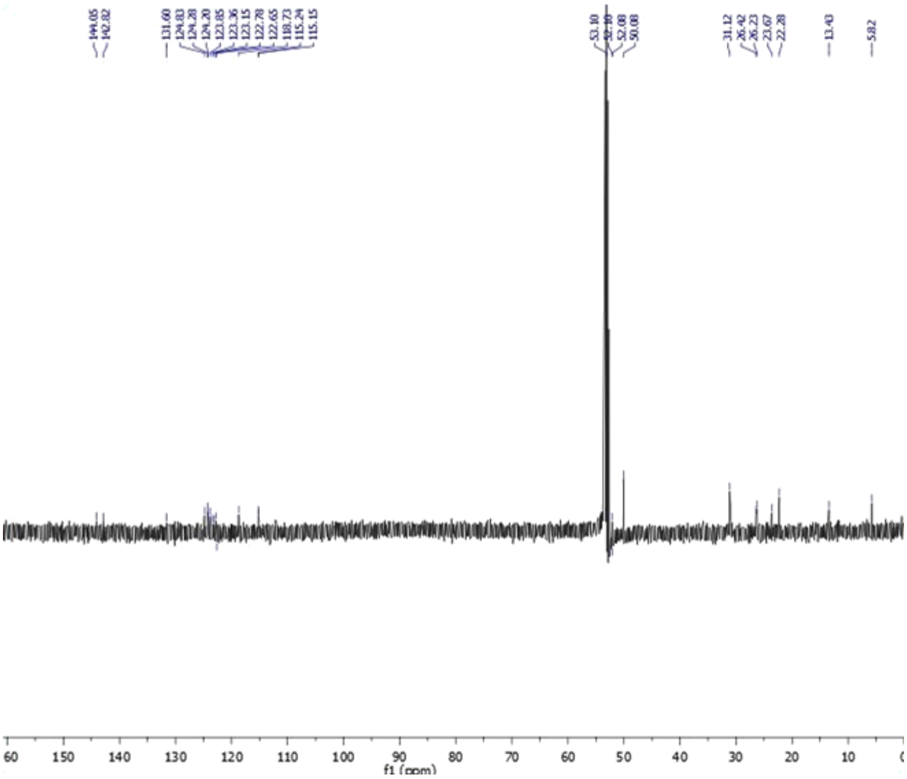

Figure S12. ^{13}C NMR spectrum of compound **3f** (125 MHz, CD_2Cl_2, RT).

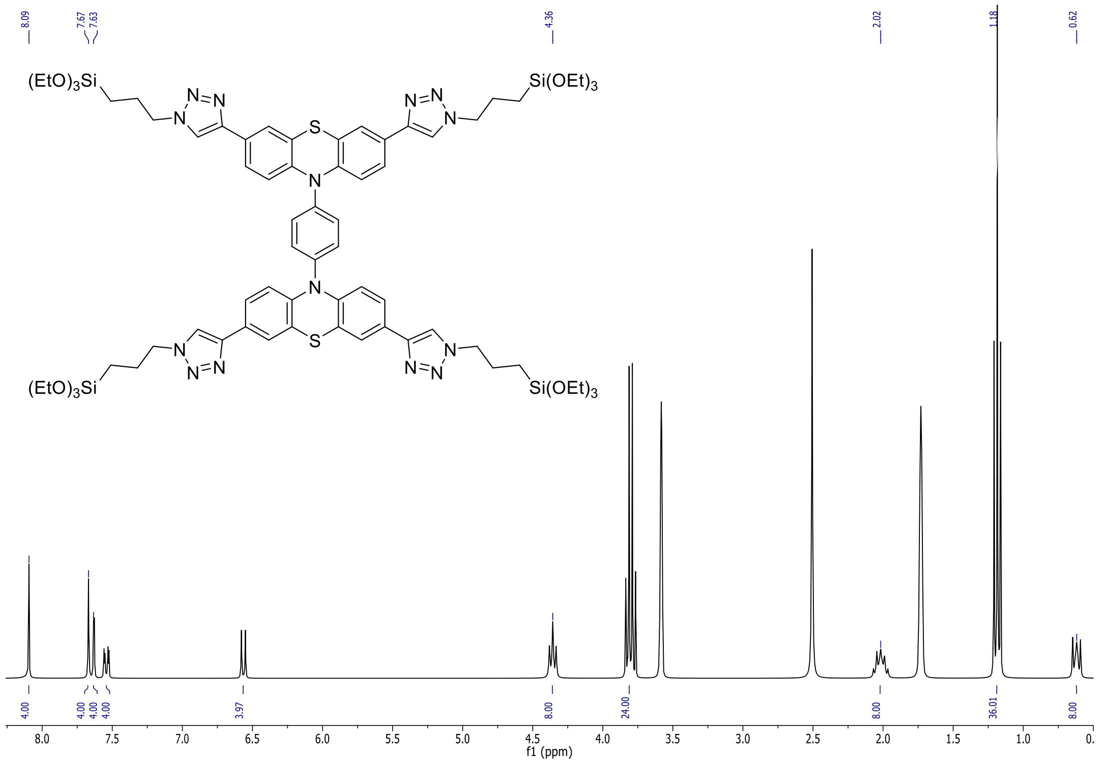

Figure S13. ^{1}H NMR spectrum of compound **8a** (300 MHz, **t**etrahydrofuran-d_8, 298 K).

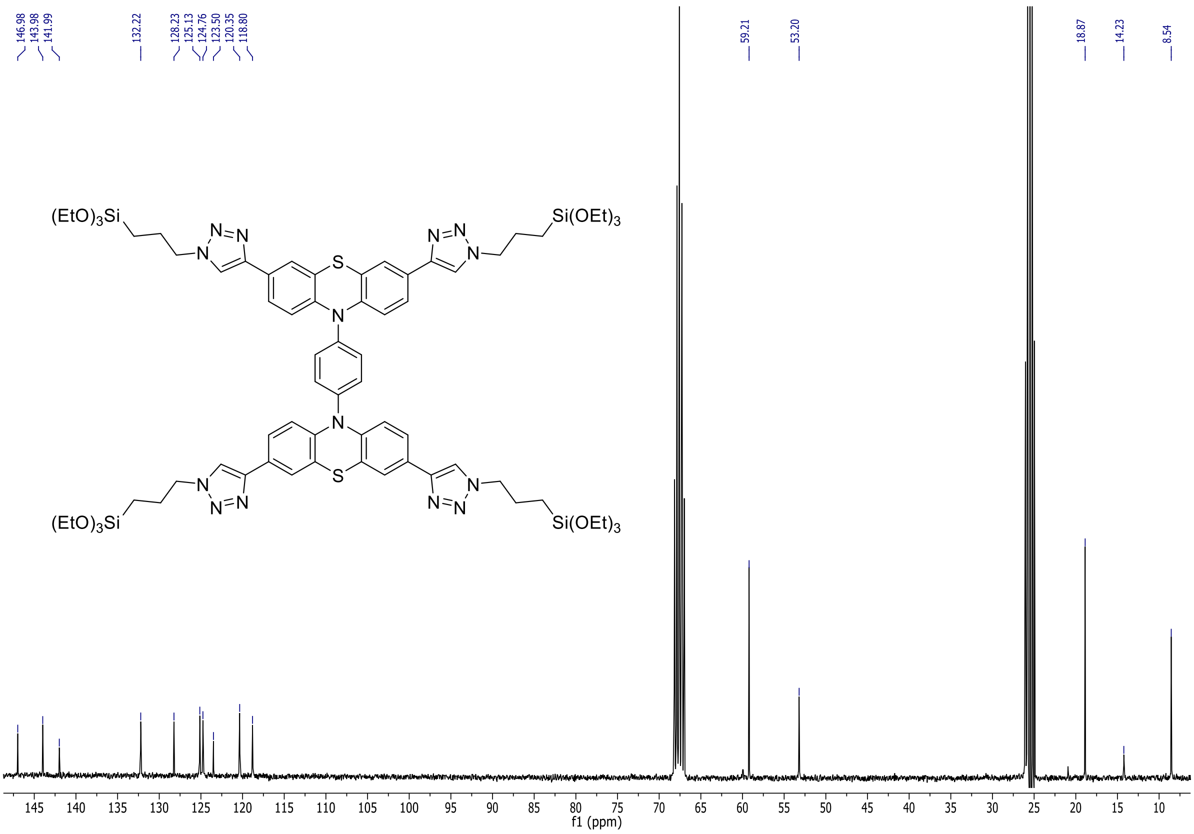

Figure S14. ^{13}C NMR spectrum of compound **8a** (75 MHz, tetrahydrofuran-d_8, 298 K).

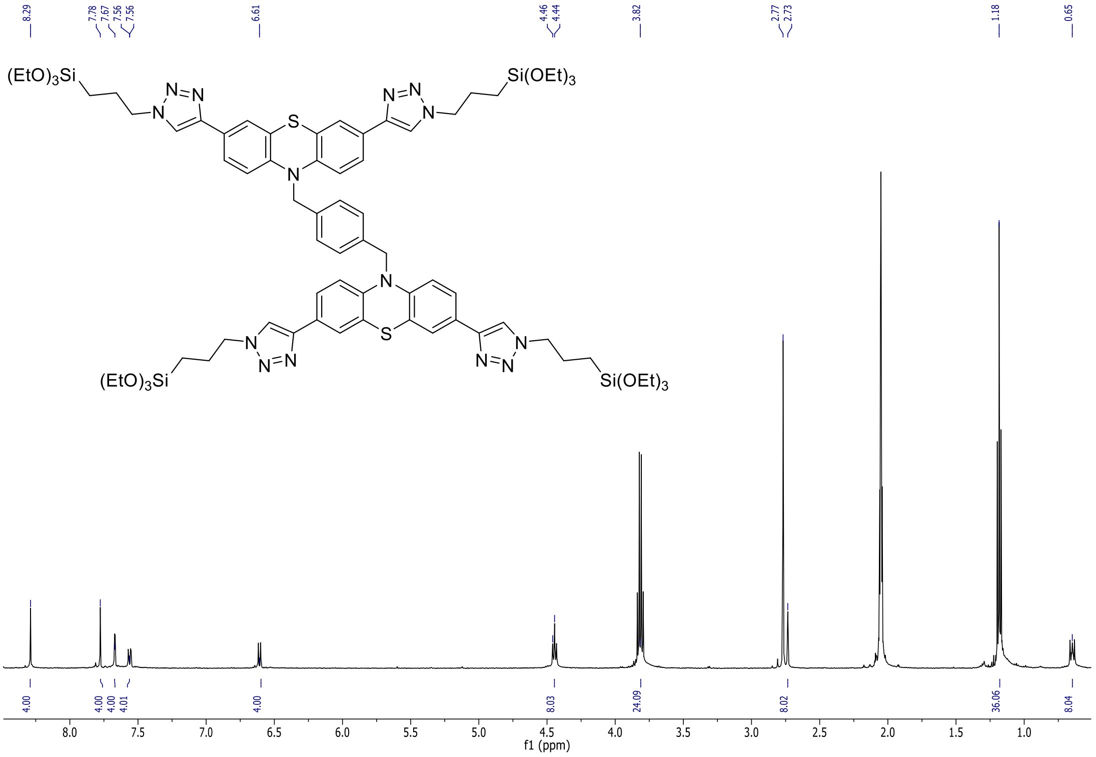

Figure S15. ^{1}H NMR spectrum of compound **8b** (500 MHz, acetone-d_6, 298 K).

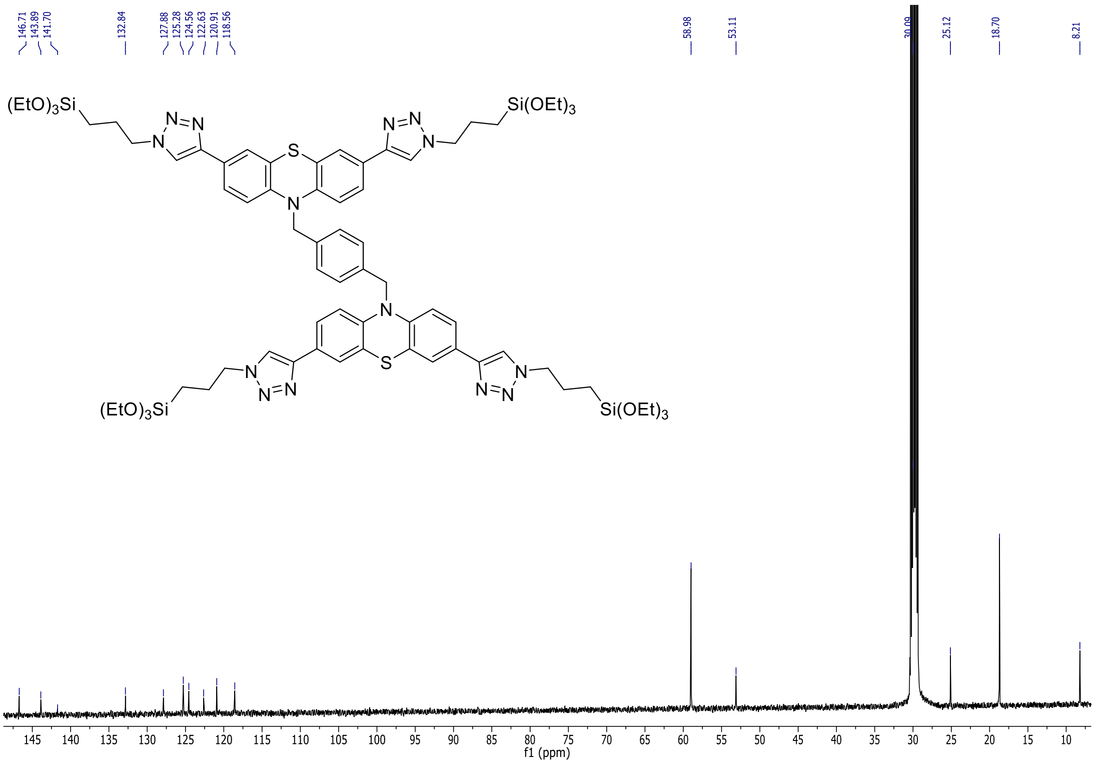

Figure S16. ^{13}C NMR spectrum of compound **8b** (125 MHz, acetone-d_6, 298 K).

2. Textural parameters of 3b-d@MCM-41

Table S1. Textural parameters of **3b-d@MCM-41** derived from and powder X-ray diffraction and nitrogen adsorption-desorption analyses with the phenothiazine loading in the corresponding hybrid materials.

sample	d_{100}[a] [nm]	a_0[b] [nm]	S_{BET}[c] [m^2g^{-1}]	V_p[d] [cm^3g^{-1}]	D_p[e] [nm]	w_t[f] [nm]	content of phenothiazine[g] [mmol g^{-1}]	[wt%]
**3b@MCM-41**	4.20	4.85	536	0.36	2.36	2.49	0.70	30.4
**3c-1@MCM-41**	4.44	5.13	1086	0.96	2.82	2.31	0.28	18.3
**3c-2@MCM-41**	4.50	5.20	543	0.48	2.77	2.43	0.43	28.1
**3d@MCM-41**	4.48	5.17	539	0.41	2.41	2.76	0.28	14.9

[a]d_{100} is the d(100) spacing. [b]a_0 is the cell parameter (a_0=2d_{100}/√3). [c]S_{BET} is the BET surface area; [d]V_p is the pore volume; [e]D_p is the pore diameter; [f]w_t is the wall thickness a_0-D_p; [g]Calculated from the CHN combustion analysis according to the content of nitrogen.

2.1. Powder XRD measurements and mesostructure of the hybrid materials 3b-d@MCM-41

2.1.1. Hybrid material 3b@MCM-41

The XRD pattern of **3b@MCM-41** shows that the reflection of the 100 plane of hexagonal symmetry is located at 2θ = 2.1°, which corresponds to a *d*-value of 4.20 nm (Figure S17). Additionally, an overlapping broad band can be observed in the 2θ rage of 3.4–4.8°, which should be the diffraction of the 110 and 200 planes of the hexagonally symmetric lattice of the MCM-41 type materials.[1] This result demonstrates the presence of a periodic arrangement of pores in the materials, although the diffraction peaks not very sharp and intense. The broadened signals are due to the large amount of precursor **3b** that was incorporated inside the pores of this material (0.70 mmol/g, 30.4 wt%), which decreases the contrast density between the framework and the pore channels.[2,3] Simultaneously, the stiffness of the large molecule **3b** implies a strong strain on the cylindrical channels.[4] Therefore, the structure distortion is reflected by decreased intensity and broadening of the diffraction peaks. A slight decrease of the cell parameter a_0 = 4.85 nm can is comparable to a_0 = 4.96 nm of pure silica MCM-41.[5] This phenomena may also be explained by the large amount of **3b** involved in the cocondensation synthesis, and the large size of the phenothiazine with the triazole head group,

that will disturb the inorganic silica species to fully cover the surfactant micelles. Thus after hydrothermal hydrolysis and condensation of the silica species, a shrunken lattice value can be observed.

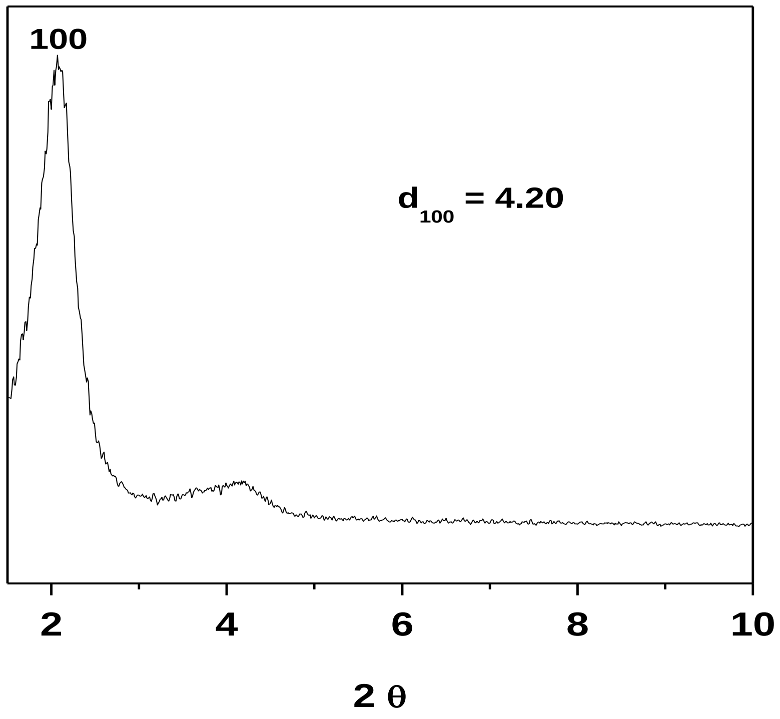

Figure S17. Powder XRD pattern of **3b@MCM-41**.

2.1.2. Hybrid materials 3c@MCM-41

At two different amounts of biphenothiazine **3c** the materials **3c-1@MCM-41** and **3c-2@MCM-41** were obtained that display some differences in powder XRD patterns (Figure S18). The sample **3c-1@MCM-41** with a loading of 0.28 mmol/g of biphenothiazine presents four typical distinguished Bragg diffraction peaks, which belong to the 100, 110, 200 and 210 planes. This series of peaks can be indexed to a two-dimensional hexagonal *p6mm* symmetry, which is similar to the pattern of ordered MCM-41, indicating a highly long-range ordered mesoporous structure. The d_{100} and a_0 values of **3c-1@MCM-41** (4.44 nm and 5.13 nm) are larger than the d_{100} and a_0 values of MCM-41 (4.30 nm and 4.96 nm). Upon increasing the loading of **3c** to 0.43 mmol/g, a hybrid material **3c-2@MCM-41** is obtained. The XRD pattern of this sample (Figure X, (b)) shows a relatively broad diffraction peak of 100 plane with low intensity, and an overlapping band around $2\theta = 4^{o}$ that can be assigned to the 110 and 200 plane. This apparent reduction in diffraction intensity and increase in peak width are often observed, caused by a

large amount of organic species inside the pores. These large size organic molecules either distort the pore structure, or decrease the contrast matching between framework and channel. Additionally, the peaks of **3c-2@MCM-41** are shift to lower angle (diffraction of 100 phase) compared to the peaks of **3c-1@MCM-41,** which corresponds to a larger d_{100} (4.50 nm) and a larger a_0 (5.20 nm).

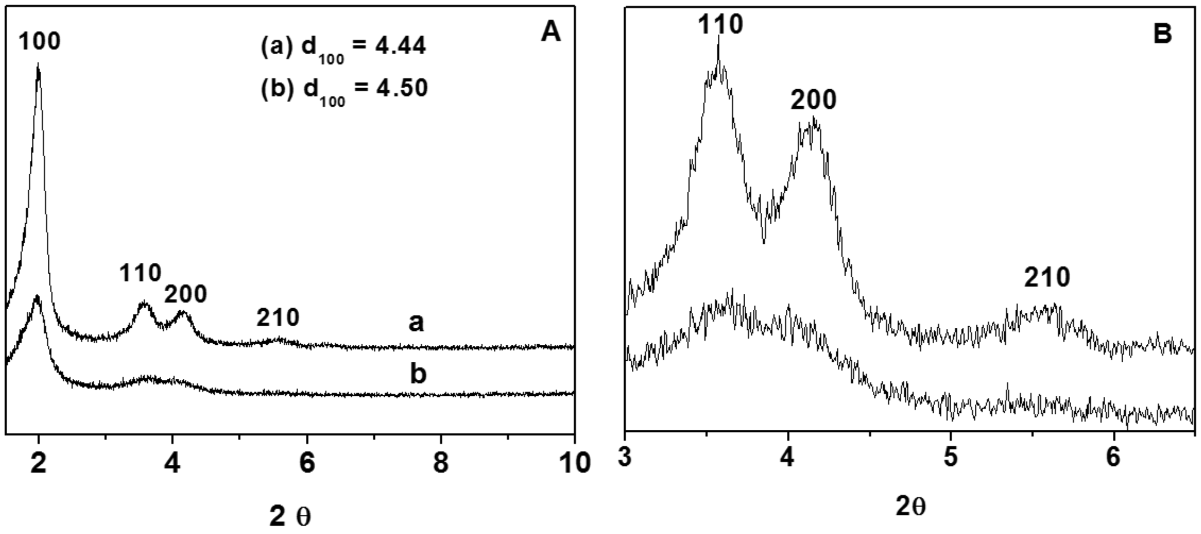

Figure S18. A: Powder XRD pattern of (a) **3c-1@MCM-41** and (b) **3c-2@MCM-41**; B: magnified signals at higher angle of (a) and (b).

2.1.3. Hybrid material 3d@MCM-41

The periodic structure of the template free mesoporous solid **3d@MCM-41**, obtained from a template supported cocondensation of the bridged organosilsesquioxane precursor **3d** with TEOS, was also determined by powder X-ray diffraction (Figure S19). It presents a Bragg reflection at about $2\theta = 2^{o}$, which confirms the formation of an ordered mesoporous structure with an interplanar *d* spacing of 4.48 nm and an approximately uniform pore sizes. An overlapping and indistinct reflection in the low-angle region can be observed, indicating limited periodicity in the long-range order of the mesoporous system. This type of diffraction is similar to the mesostructure of the material with a wormhole-like pore system (or HMS silica phases).[6] The decrease of periodicity in this solid is most likely due to the size and the geometry of the precursor **3d**, which perturbs the micelles in the original sol. Hence, a disturbed crystallization

process of the inorganic species is anticipated cycling with the less ordered micelles to obtain less ordered mesoporous material.

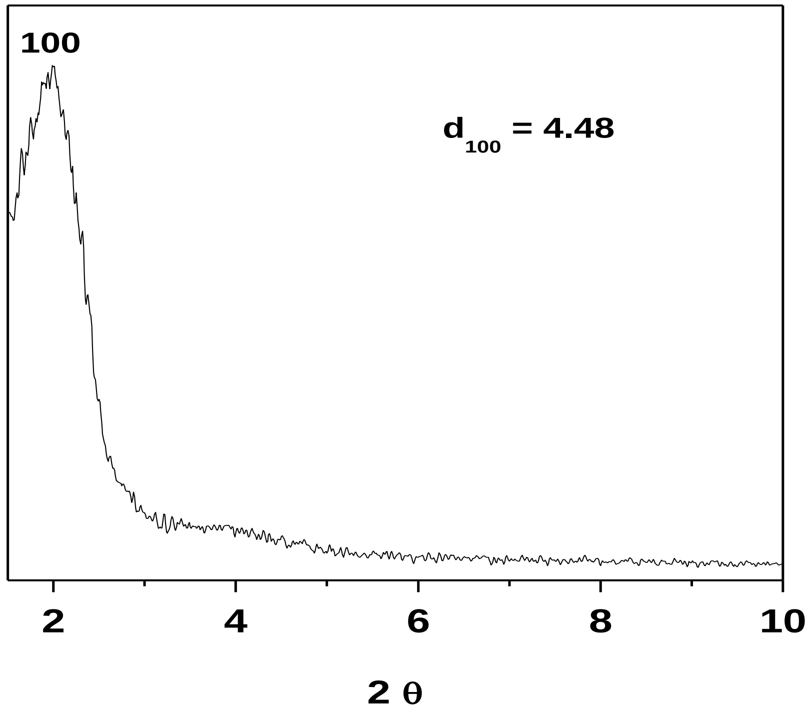

Figure S19. Powder XRD pattern of **3d@MCM-41**.

2.1.4. N_2 adsorption/desorption isotherms and pore structure of the hybrid materials 3b-d@MCM-41

Hybrid material 3b@MCM-41

The N_2 adsorption desorption isotherm of **3b@MCM-41**, corresponds to the profile of a type-IV isotherm according to IUPAC nomenclature[7] indicating the mesoporous property of this hybrid material (Figure S20A). Furthermore, a sharp capillary condensation implies a uniform porous material confirmed by the pore size distribution curve (Figure S20B). From the desorption branch of the isotherm, a mean pore size of 2.36 nm was estimated by the BJH method combining with a specific surface area 536 m^2/g, calculated by the BET method. This indicates the basic characteristics of the ordered mesoporous material. An apparent H4 type of hysteresis loop on the high pressure portion of isotherm can be indexed to uniform slit-shaped pores, which may be due to aggregates of small particles or to connections between cylindrical channels[8,9] This hysteresis loop is corresponding to the small peak around 3.80 nm in the pore size distribution curve (Figure S20B).

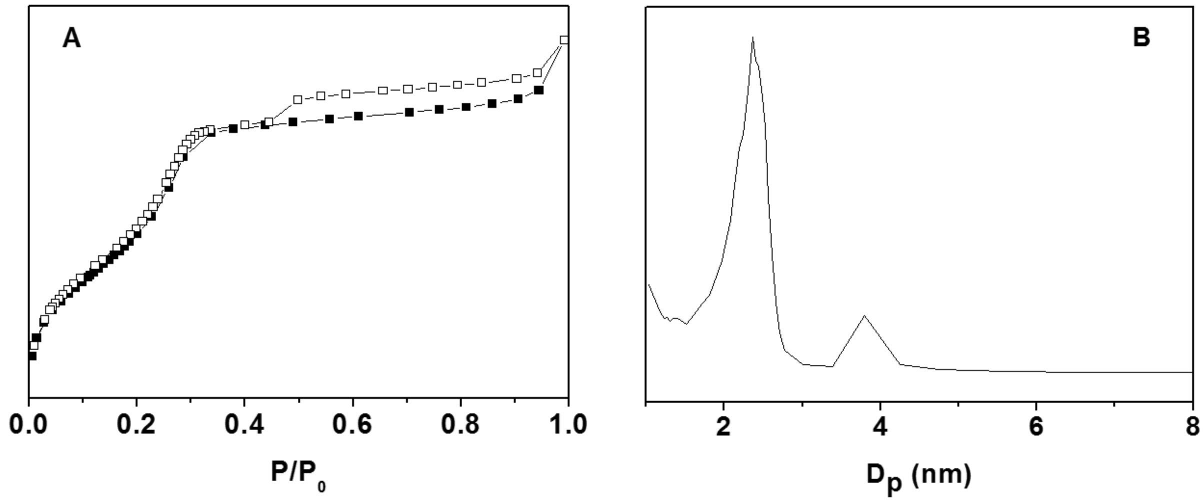

Figure S20. A) N_2 adsorption/desorption isotherms of **3b@MCM-41** (adsorption points are marked by solid squares and desorption points by empty squares). B) Pore size distribution curves (deduced from the desorption data of the isotherms).

Hybrid material 3c@MCM-41

The isotherms of the two samples of **3c@MCM-41** differ in the organic loading (**3c-1@MCM-41**: 0.28 mmol/g; **3c-2@MCM-41**: 0.43 mmol/g), but both show typical type-IV isotherms, which is a main feature of ordered mesoporous materials with a well-defined capillary condensation step at p/p_0 0.28 and 0.24, respectively (Figure S21A). The shift of the condensation point from **3c-1@MCM-41** to **3c-2@MCM-41** indicates a decrease in pore size caused by increasing the percentage of the organic moiety in the material. Accordingly, a reduction to a lower nitrogen uptake demonstrates the decrease of surface area, which again corresponds with an increase of material density with an increased loading of phenothiazine. Additionally, the curves of the pore size distribution (PSD) determined by the BJH method from the data of desorption branches of the isotherms indicate a slight decrease in the pore size by increasing of organic loading (Figure S21B), which is committed by a significant deduction of the pore volume from 0.95 to 0.48 cm^3/g (Table 2 in the manuscript). However, both of the samples preserve a narrow pore size distribution, demonstrating the uniform pores of these materials.

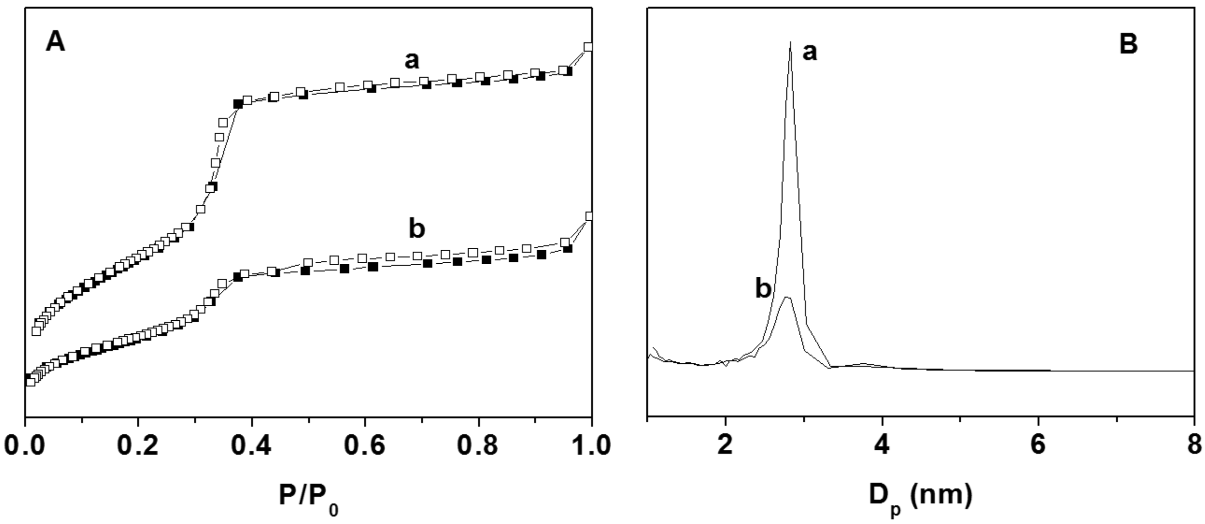

Figure S21. A) N_2 adsorption/desorption isotherms of (a) **3c-1@MCM-41**, and (b) **3c-2@MCM-41** (adsorption points are marked by solid squares and desorption points by empty squares). B) Pore size distribution curves of (a) **3c-1@MCM-41**, and (b) **3c-2@MCM-41** (deduced from the desorption data of the isotherms).

Hybrid material 3d@MCM-41

The adsorption-desorption isotherm of **3d@MCM-41** is again characterized by a sharp capillary condensation step (p/p_0 close to 0.2) and indicates a uniform mesoporous material (Figure S22A). Simultaneously, a type II uptake near the end section of the isotherm with a H4 type of hysteresis loop observed at higher partial pressures can be attributed to interstitial cavities, which may be created by a textural porosity within the particles of the materials, or an intersecting disorder of the materials.[8,9] A narrow pore-size distribution curve (Figure S22B) with a mean pore size of 2.41 nm, calculated from the adsorption branch of the isotherm using the BJH algorithm, confirms the uniformity of the material's mesopores. Another larger pore mainly located at 3.80 nm is deduced from the uptake of the condensation step at p/p_0 0.44. It might be due to the presence of intersecting pores between the uniform particles. This material possesses a relatively high specific surface area of 539 m^2/g (Table 2 in the manuscript).

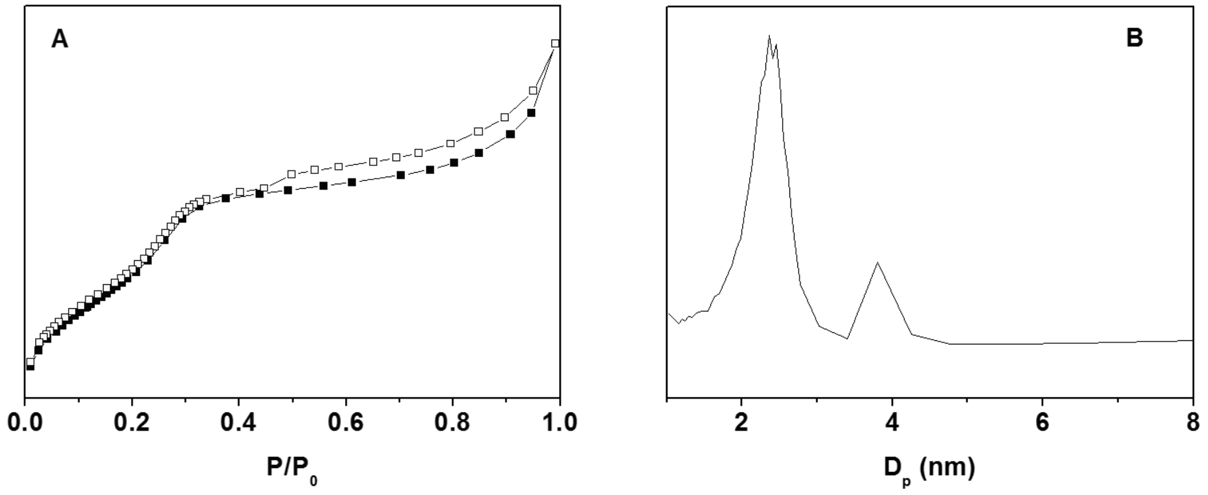

Figure S22. A) N_2 adsorption/desorption isotherms of **3d@MCM-41** (adsorption points are marked by solid squares and desorption points by empty squares). B) Pore size distribution curves of **3d@MCM-41** (deduced from the desorption data of the isotherms).

2.2. Electron microscopy of hybrid materials 3b-d@MCM-41

Transmission electron microscopy (TEM) was used to investigate the mesoporous structure of these hybrid materials.

2.2.1. TEM of 3b@MCM-41

In the TEM image of **3b@MCM-41** (Figure S23) the mesoscale pore can directly be observed in the large area from [10] direction (Figure S23A) and the parallel channel can be seen in some areas from [11] direction (Figure S23B). However, instead of a hexagonal structure of highly ordered mesopores, the existence of relatively large areas of dislocations and disclinations[10] in the mesophase of this hybrid material is obvious. This might be caused by the presence of a large amount of the large sized molecule hanging on the internal wall of pore, which exerts strain on the wall to distort the pore shape. This structural defect involves a large strain field (the bend channels) and the large bend will break channels into many short sections in some area. Meanwhile, this TEM result is coherent to the pattern of XRD, where a type of twisted arrangement of channels is present in this material, instead of the highly ordered hexagonal array.

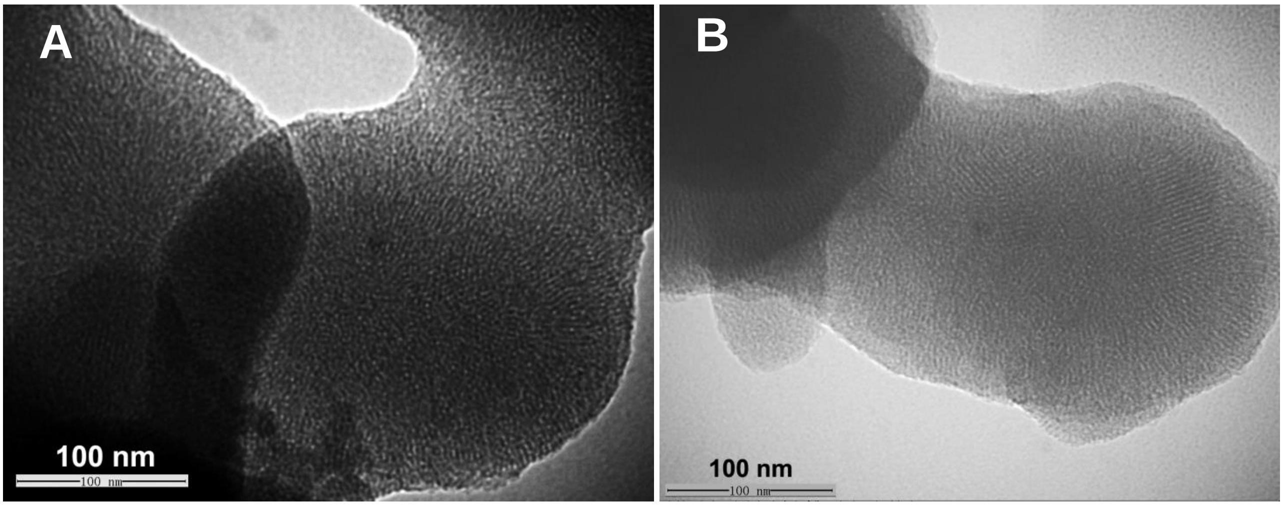

Figure S23. TEM images of **3b@MCM-41**. A) Large area in [10] direction. B) Parallel channels discernable in some areas in [11] direction

2.2.2. TEM of 3c@MCM-41

The TEM images of the both samples of **3c@MCM-41** are depicted in Figure S24. A TEM analysis of **3c-1@MCM-41** revealed ordered arrays of cylindrical channels arranging along the [11] direction and demonstrates a highly ordered pore structure (Figure S24B). This type of ordering are even discernable in the large area image (Figure S24A) indicating the long-range order of this hybrid material as expected form the distinct diffraction pattern of the XRD. The image of **3c-2@MCM-41** in [10] direction (Figure S24C) displays a distorted pore structure implying the reduced overall order of this material. However, a relatively ordered channel arrangement becomes apparent in the perpendicular direction (Figure S24D) proving the existence of a mesoporous structure with a slight decrease from ordered orientation. This is according to the result of XRD and it is often observed for hybrid materials with a high organic loading.

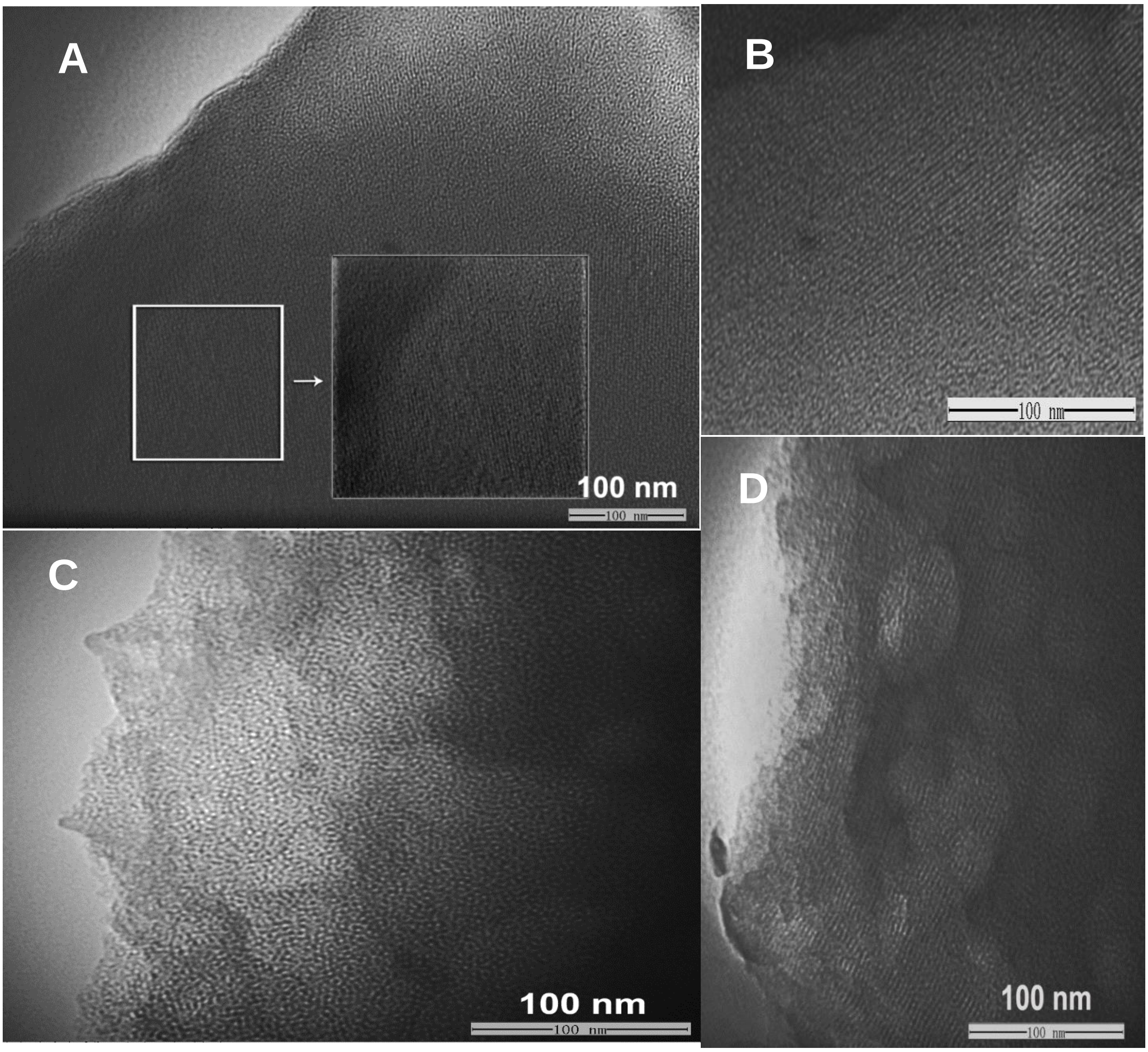

Figure S24. TEM images of **3c@MCM-41**. A) Large scale image of sample **3c-1@MCM-41**. B) Image perpendicular to the channel direction of sample **3c-1@MCM-41**. C) Image of sample **3c-2@MCM-41** along the channel direction. D) Image of sample **3c-2@MCM-41** perpendicular to the channel direction.

2.2.3. TEM of 3d@MCM-41

The TEM images of **3d@MCM-41** (Figure S25) show the presence of wormhole-like structures rather than a 2D hexagonal mesostructure as suggested by the XRD pattern. Thus, the mesostructuring of this material occurs in the range between unordered compounds and highly ordered periodic mesoporous organosilicas. This relative disordered structure might be caused by the limitation of wall thickness of MCM-41 type of materials, which are not sufficient for

maintaining such large organic molecules. Thus, an alternative embedding of this molecule into a thicker wall material, such as SBA-15, might be considered.

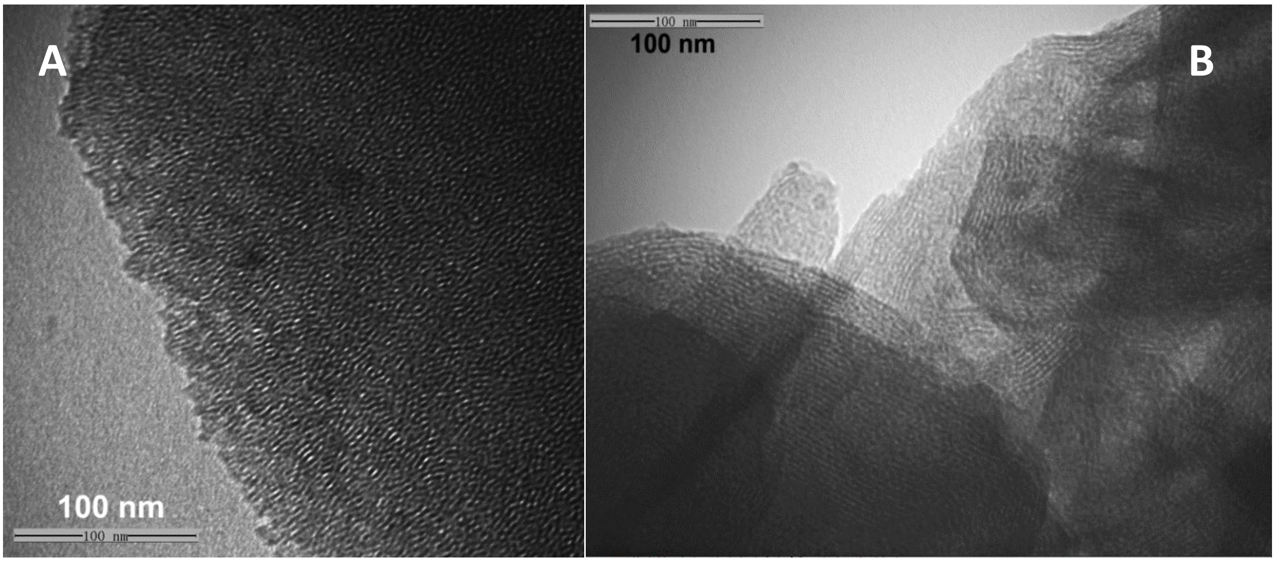

Figure S25. TEM images of **3d@MCM-41**. A) Along the channel direction. B) Perpendicular to the channel direction.

The morphology of the mesoporous materials can be influenced the synthetic conditions. Unpredictable effects in the synthesis of hybrid mesoporous materials by the co-condensation strategy originate from the concentration of the organosilane precursor and the molecular structure of this precursor. As previously shown for urea linked phenothiazine precursors a change the morphology of final product can be adjusted by the concentration and the molecular structure.[5] As seen in the SEM (scanning electron microscopy) image of **3b@MCM-41** (Figure S26) the triazole linked precursor can also be responsible for a morphology change. An aggregation of a large amount of small spherical particles are discernable. From the magnified cutout in Figure S26, the particles located inside this agglomerate have a smoother surface than the outside ones. In more detail, many small particles gather on the outside particles forming a rough surface. The assembly of small particles can be observed in this material, which is coherent with the analysis from the isotherm of N_2 adsorption desorption experiment.

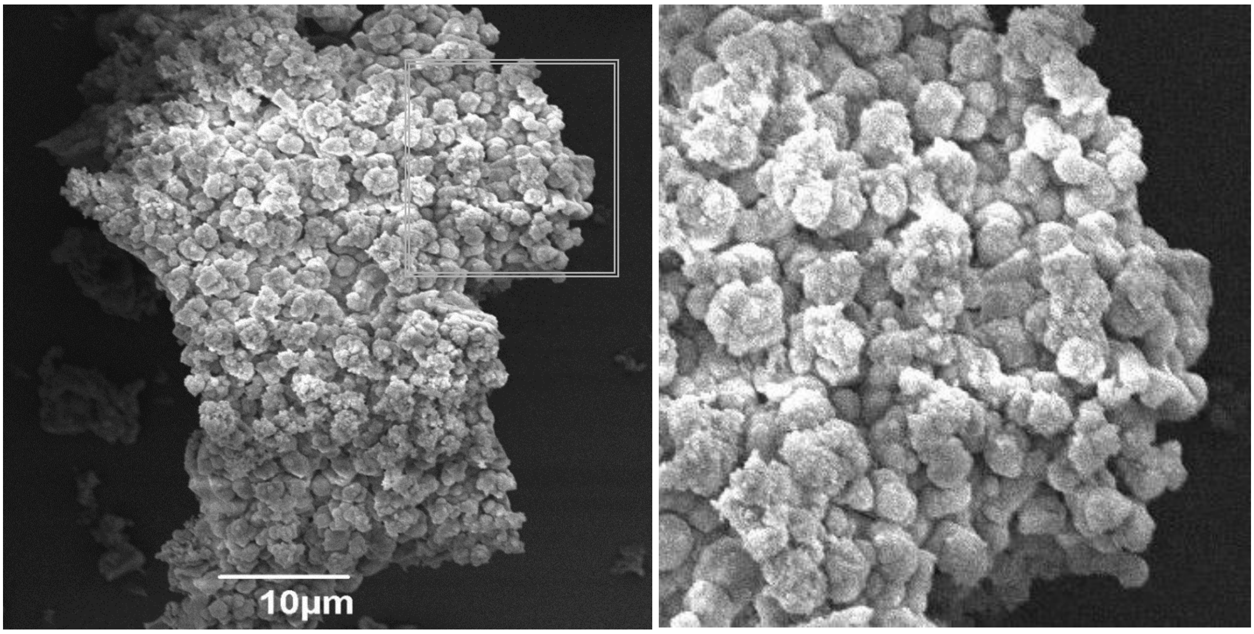

Figure S26. SEM image of hybrid mesoporous materials **3b@MCM-41** (right) and magnified cutout (right).

2.3. Spectroscopic characterization of hybrid materials 3b-d@MCM-41

2.3.1. FT-IR spectra of hybrid materials 3b-d@MCM-41

The FT-IR spectra are depicted in Figure S27. The presence of the triazole linked phenothiazine is clearly seen in the FT-IR spectrum of **3b@MCM-41** (Figure S27A). The C-H stretching vibration of methyl or methylene groups in the alkane chains causes the peaks around 2980-2820 cm^{-1}. The strong peak at 1465 cm^{-1} is attributed to the C-H deformation of the CH_2 group or the antisymmetric deformation of the CH_3. In addition to these significant characteristic signals of the organic moiety, the spectra of all hybrid materials also show some distinct feature peaks of the silica framework. A broad band in the range of 3600-3200 cm^{-1} can be assigned to the ν_{OH} stretching vibration of the hydrogen bonded internal silanol groups.[11] The broad bands at 1300 to 1100 cm^{-1} are attributed to the asymmetric stretching vibration of various Si-O-Si bonds of the silica network. The band at 955 cm^{-1} is attributed to the stretching vibration of surface Si-O^- groups, and the peak at 800 cm^{-1} is attributed to the symmetric vibration of Si-O-Si. The strong peak at 460 cm^{-1} can be assigned to the bending band of Si-O-Si.[12]

According to the FT-IR spectra of **3c-1@MCM-41** and **3c-2@MCM-41** (Figure S27B) the C-H stretching vibration (2980-2820 cm^{-1}) is more significant than for **3b@MCM-41** due to the presence of the biphenothiazine moieties with two hexyl chains. In addition, the relative intensity of the sharp signal of the C-H deformation vibration at 1460 cm^{-1} is increased. The

FT-IR spectrum of **3d@MCM-41** (Figure S27C) shows the same expected features stemming from the phenothiazinyl core.

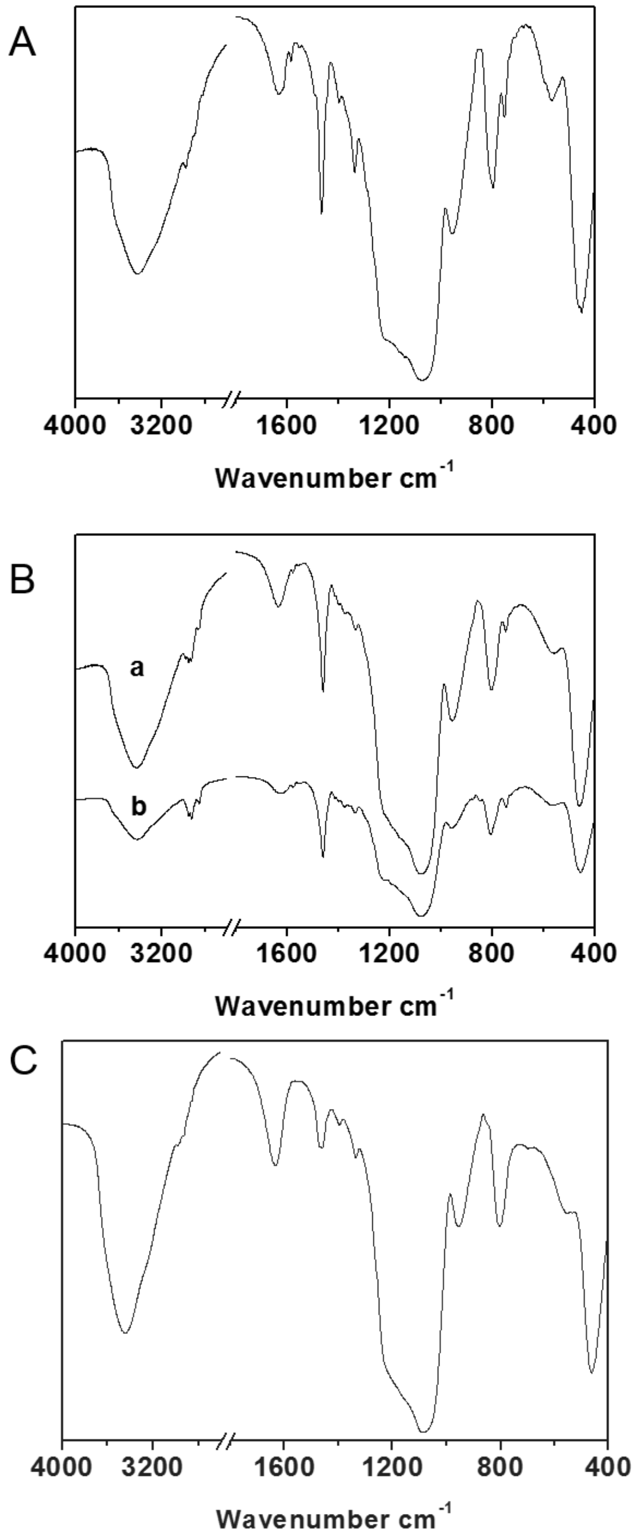

Figure S27. FT-IR spectra of the hybrid materials **3b@MCM-41** (A), **3c-1@MCM-41** (B-a), **3c-2@MCM-41** (B-b), and **3d@MCM-41** (C).

2.3.2. ^{13}C CP MAS NMR spectra of hybrid materials 3b-d@MCM-41

The molecular integrity of the phenothiazine moieties and ligation inside the silica material by covalent bonds in the hybrid materials **3b-d@MCM-41** is additionally supported by solid-state NMR spectroscopy (Figure S28). The ^{13}C CP-MAS NMR spectrum of **3b@MCM-41** exhibits signals (Figure S28A), which match with the high resolution spectrum of precursor **3b** in solution (red lines). This indicates that the organic moieties are not disintegrated during the hydrothermal synthesis and the acidic extraction process. The resonances in the high field region predominantly stem from the carbon nuclei of the hexyl chain, while there are still residual amounts of Si-OCH_2CH_3 groups or some ethanol from the template extracting process (singlets at ~ δ 60 and ~ δ 16). An important resonance at ~ δ 52 is assigned to the -CH_2-N group linked to the triazole. The signals in the range from δ 115 to 132 are assigned to the carbon nuclei of the electronrich phenothiazine ring and the triazole ring.

Likewise in the ^{13}C CP MAS NMR spectrum of **3c-2@MCM-41** (Figure S28B), the resonances of supported material are consistent to the peaks of the organic precursor **3c** (red lines), indicating the integrity of precursor **3c** in this material. The intense resonances in the low field region are mainly attributed to carbon nuclei of the two phenothiazine cores. The preservation of a signal at ~ δ 52 ppm corresponds to the carbon of the CH_2-N group, which indicates that the ligation between the propyl and triazole group remained uncleaved. Other peaks in the high field region can be assigned to the carbon atoms of the hexyl side chain.

The solid-state ^{13}C NMR data of **3d@MCM-41** (Figure S28C) with the matching between the solid material and the solution spectrum of precursor **3d** also proves the successful integration of the precursor in the solid material. Asterisks assign small amounts of remnant template.

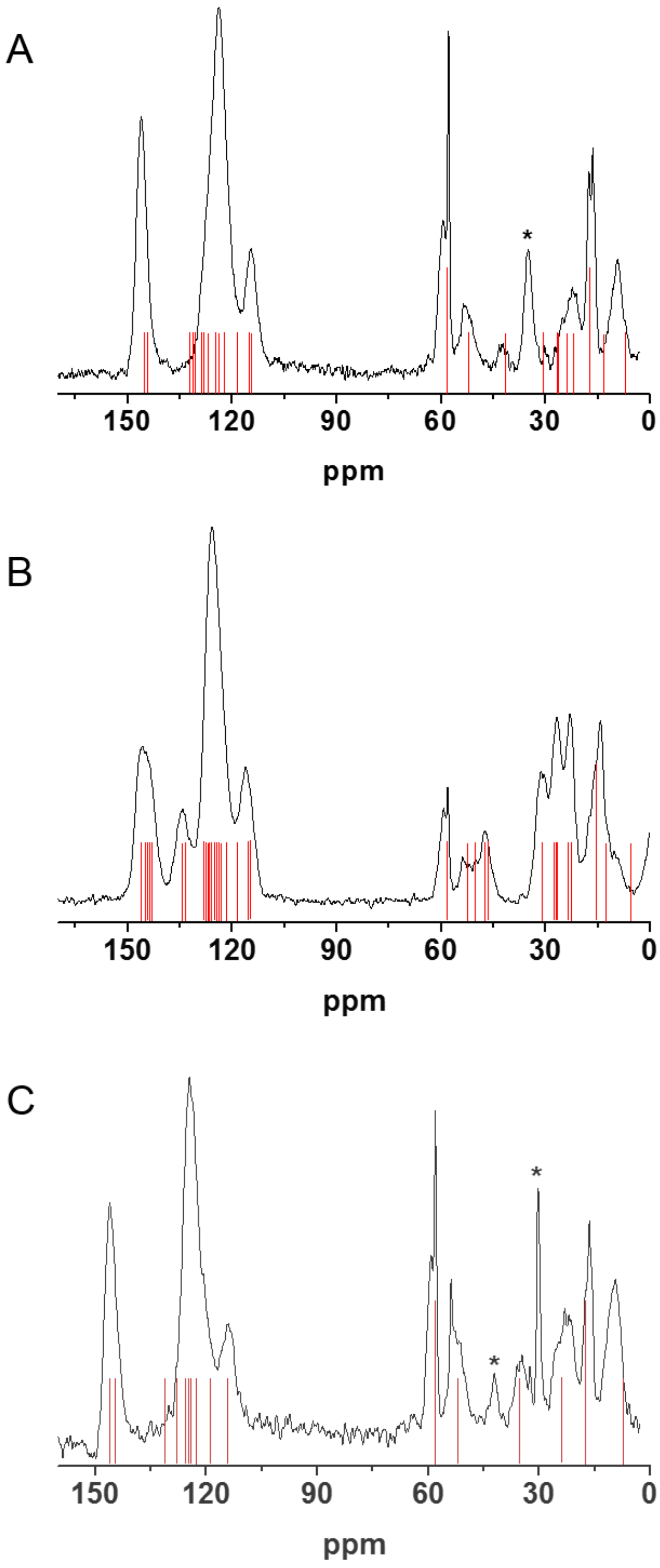

Figure S28. ^{13}C CP-MAS NMR spectra of the hybrid materials **3b@MCM-41** (A), **3c-2@MCM-41** (B), and **3d@MCM-41** (C). The inserted red lines show the positions and relative intensities in the solution NMR spectra of the corresponding precursor **3**. Signal marked with an asterisk are assigned to a small amount of remnant template.

2.3.3. ^{29}Si CP MAS NMR spectra of hybrid materials 3b-d@MCM-41

The ^{29}Si CP-MAS NMR spectra confirm that the organic/inorganic moieties are the basic structure units of the hybrid mesoporous materials (Figure S29). The spectrum of **3b@MCM-41** (Figure S29A) displays two signals in the T region, indicating the presence of organosilica T^3 species [C-Si(OSi)$_3$] (δ –67) and T^2 species [C-Si(OSi)$_2$(OH)] (δ –57). The dominant integral of these two peaks, where T^3 presents the major substructure, supports a sufficient hydrolysis/condensation of precursor **3b** during the synthesis to achieve a strong linkage (three Si-O-Si covalent bonds) between the organic compound and the silica matrix. For the characteristics of inorganic silica framework, the spectrum exhibits a small signal at δ –92, which can be assigned to the Q^2 [(SiO)$_2$Si(OR)$_2$] species, a sharp and high intense signal around δ –101, which is the region of Q^3 [(SiO)$_3$Si(OR)] (R = H, Me) as the majority, and another peak is present at δ –110 ppm corresponding to Q^4 [(SiO)$_4$Si] species.[13]

The ^{29}Si CP MAS NMR spectrum of **3c-2@MCM-41** (Figure S29B) similarly displays a dominating T^3 sites signal can be observed at δ –68, indicating that the organosilica is mainly present as a C-Si(OSi)$_3$ species in this hybrid material. The maintaining of a large amount of isolated Si-OH groups is proven by the sharp peak at δ –100 (Q^3). Furthermore, the small resonance at δ –90 implies the residual geminal silanol sites.

The bridging organosilane precursor **3d** forms material **3d@MCM-41** which exhibits the ^{29}Si CP MAS NMR spectrum (Figure S29C) that T^n band signals are mainly centered around δ –68 with a small shoulder around δ –57. This type of resonances discloses the relatively complete hydrolysis of the alkyoxysilanes of this precursor. Thus, it indicates that this molecule is embedded in the framework of the hybrid material with two bonded silicon atoms.

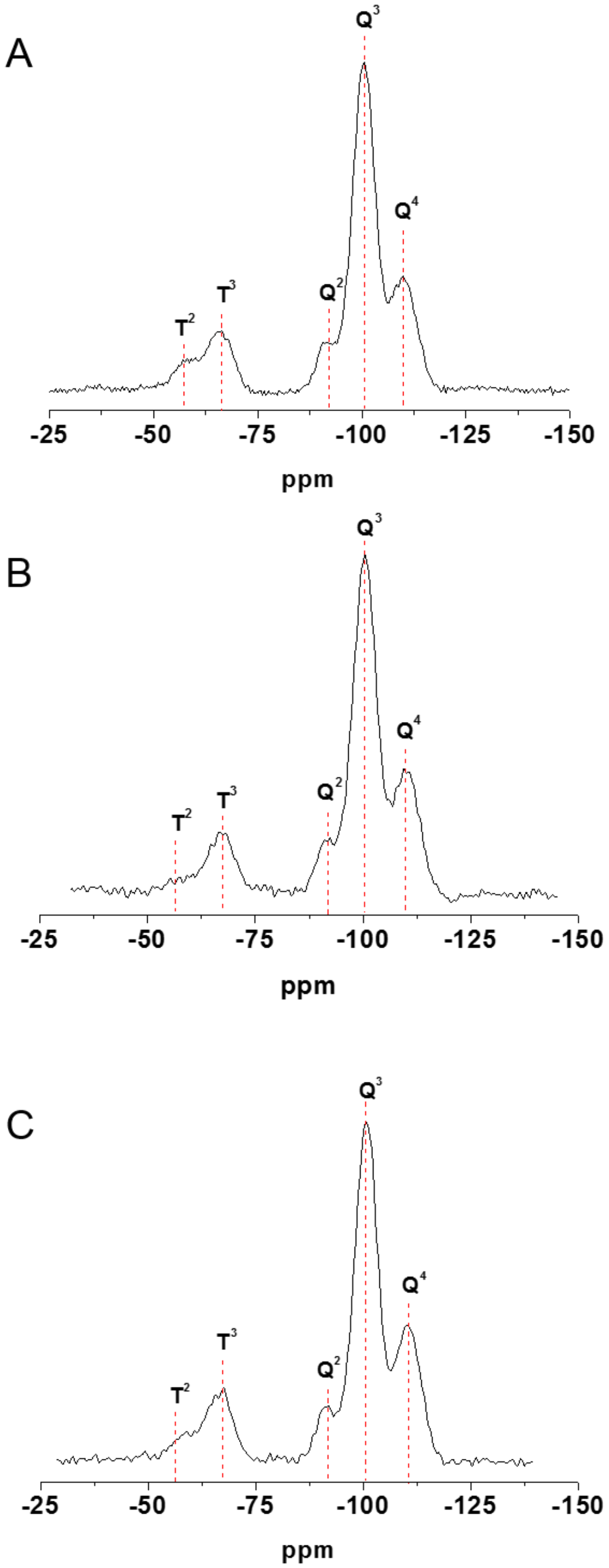

Figure S29. ^{29}Si CP-MAS NMR spectra of the hybrid materials **3b@MCM-41** (A), **3c-2@MCM-41** (B), and **3d@MCM-41** (C).

2.4. Optical Properties of Hybrid Materials 3b-d@MCM-41 and Selected Oxidized Materials

The optical spectra of hybrid materials **3b-d@MCM-41** (UV/Vis and fluorescence) as well as of their oxidation products (UV/Vis) are collected in Figures S30-S34.

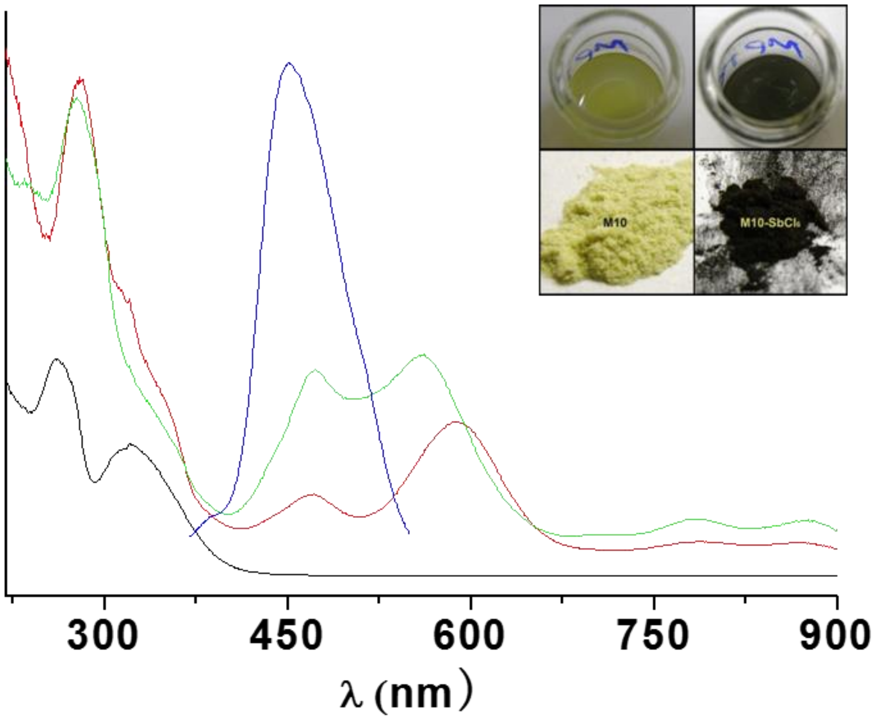

Figure S30. Superimposed electronic spectra of dichloromethane suspensions of the hybrid mesoporous material **3b@MCM-41** and its oxidized species. Absorption spectra of **3b@MCM-41** (black curve), radical cationic samples **3b$^+$@MCM-41 BF$_4^-$** (red curve), **3b$^+$@MCM-41 SbCl$_6^-$** (green curve), and the emission spectrum of **3b@MCM-41** (blue blue). The inset illustrates the color change caused by the oxidation of **3b@MCM-41** using $SbCl_5$ in suspension (top) and of the isolated corresponding solid (bottom).

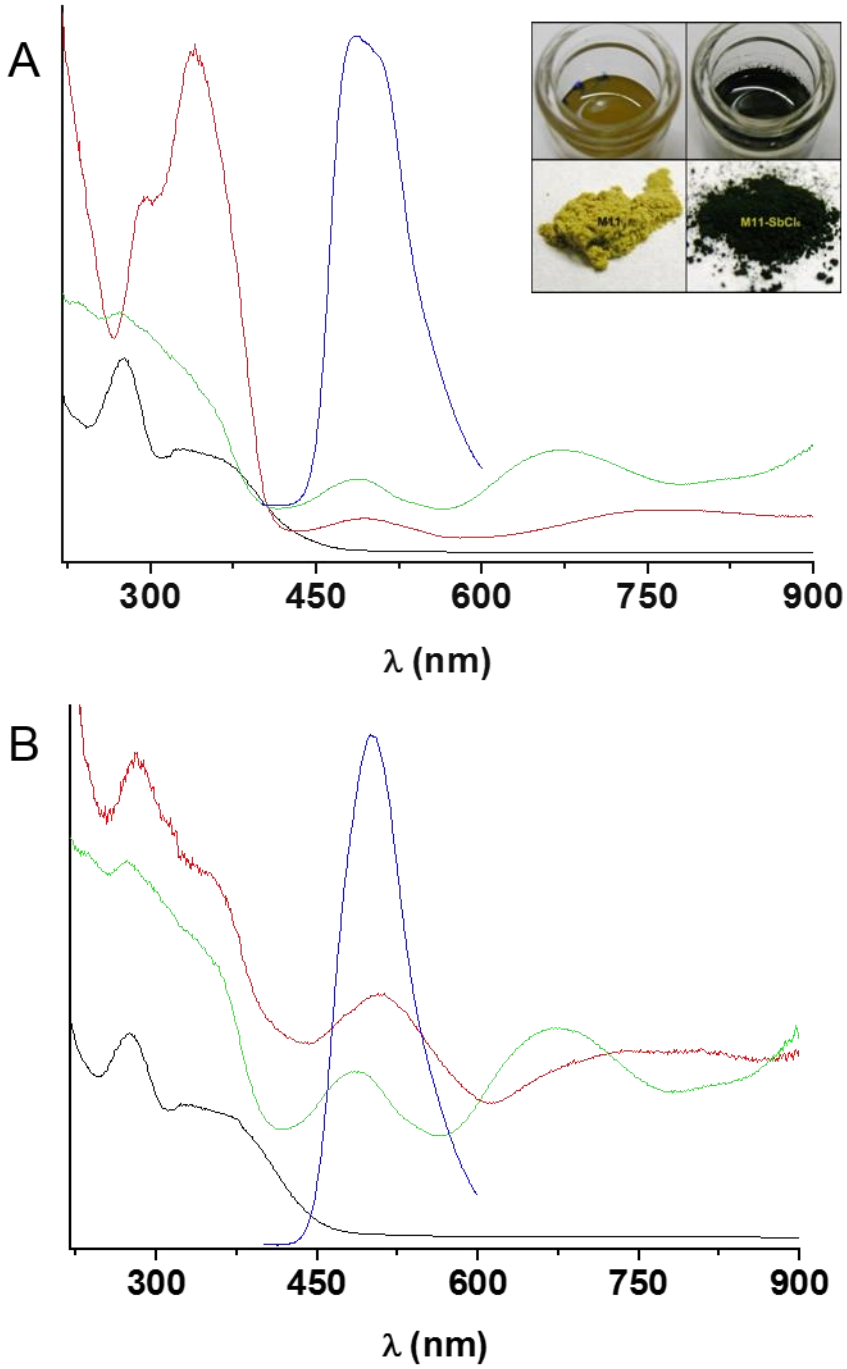

Figure S31. Superimposed electronic spectra of dichloromethane suspensions of the hybrid mesoporous materials **3c@MCM-41** and their oxidized species. A) Absorption spectra of **3c-1@MCM-41** (black curve), radical cationic samples **3c-1$^+$@MCM-41 BF_4^-** (red curve), **3c-1$^+$@MCM-41 $SbCl_6^-$** (green curve), and the emission spectrum of **3c-1@MCM-41** (blue blue). B) Absorption spectra of **3c-2@MCM-41** (black curve), radical cationic samples **3c-2$^+$@MCM-41 BF_4^-** (red curve), **3c-2$^+$@MCM-41 $SbCl_6^-$** (green curve), and the emission spectrum of **3c-2@MCM-41** (blue blue). The inset illustrates the color change caused by the oxidation of **3c-1@MCM-41** using $SbCl_5$ in suspension (top) and of the isolated corresponding solid (bottom).

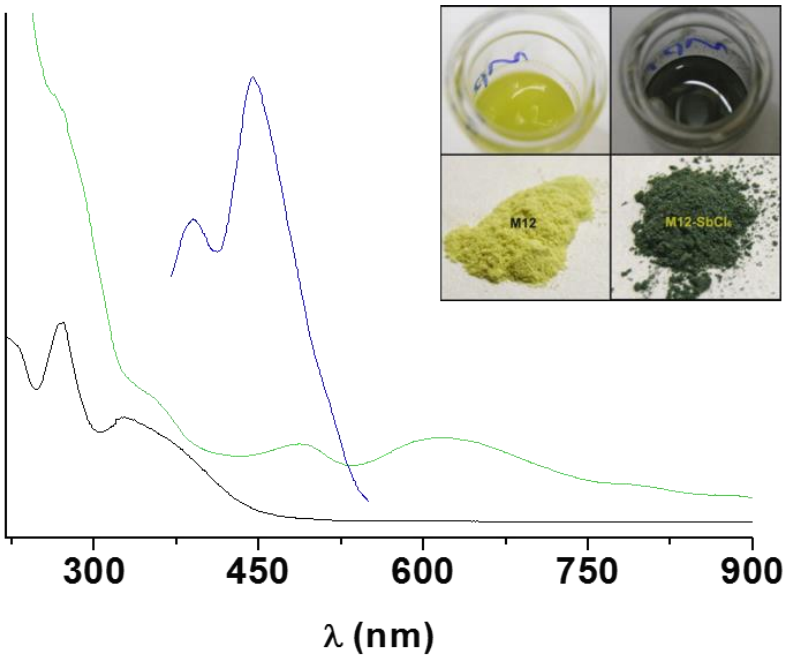

Figure S32. Superimposed electronic spectra of the dichloromethane suspensions of hybrid mesoporous material **3d@MCM-41** and its oxidized species. Absorption spectra of **3d@MCM-41** (black curve), radical cationic sample **3d⁺@MCM-41 $SbCl_6^-$** (green curve), and the emission spectrum of **3d@MCM-41** (blue blue). The inset illustrates the color change caused by the oxidation of **3d@MCM-41** using $SbCl_5$ in suspension (top) and of the isolated corresponding solid (bottom).

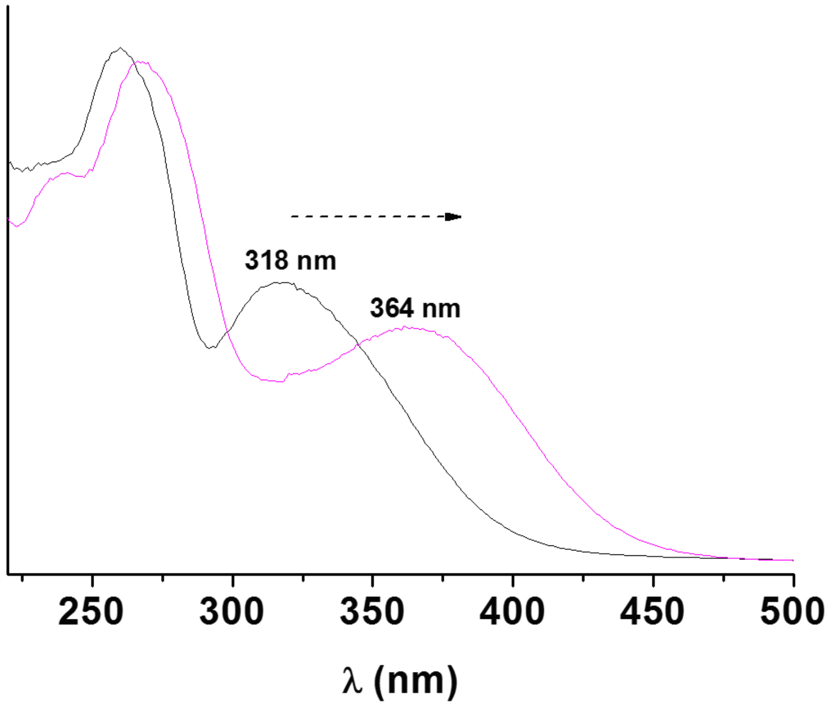

Figure S33. UV/Vis spectra of dichloromethane suspensions of the hybrid mesoporous material **3b@MCM-41** (black curve) and the corresponding protonated sample **3b@MCM-41-H⁺** (magenta curve) (protonation with trifluoroacetic acid in dichloromethane at room temp).

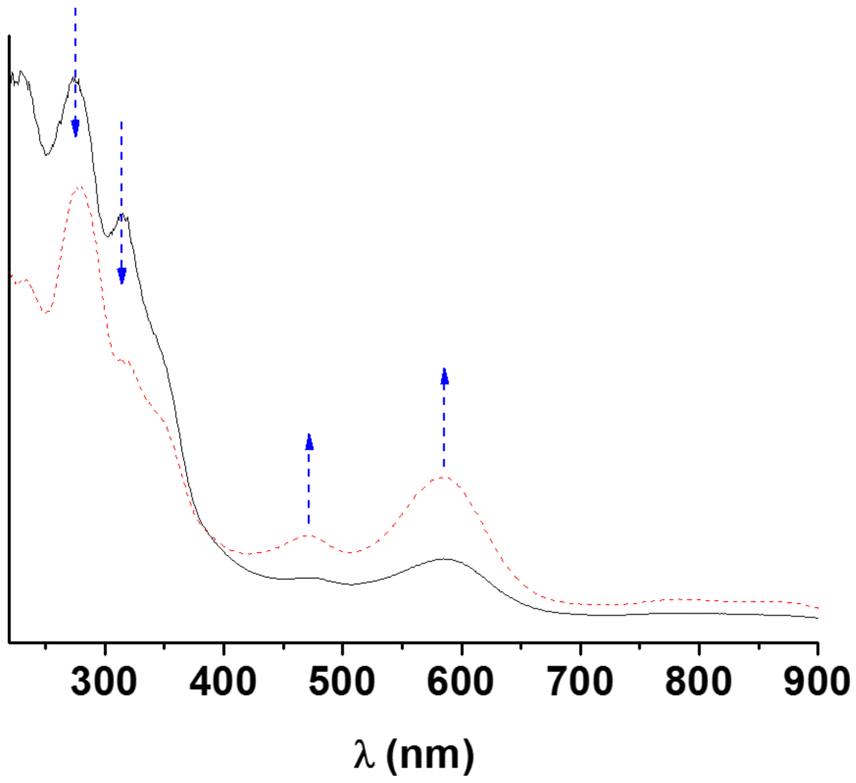

Figure S34. UV/Vis spectra of dichloromethane suspensions of the oxidized hybrid mesoporous material **3b$^+$@MCM-41 BF$_4^-$** (black curve) and the corresponding protonated sample **3b$^+$@MCM-41-H$^+$ BF$_4^-$** (red dashed curve). The arrows indicate changes of distinct absorption bands upon protonation with trifluoroacetic acid in dichloromethane at room temp.

3. References

1 Beck, J.S.; Vartuli, J.C.; Roth, W.J.; Leonowicz, M.E.; Kresge, C.T.; Schmitt, K.D.; Chu, C.T.W.; Olson, D.H.; Sheppard, E.W.; McCullen, S.B.; Higgins, J.B.; Schlenker, J.L. A new family of mesoporous molecular sieves prepared with liquid crystal templates. *J. Am. Chem. Soc.* **1992**, *114*, 10834-10843. DOI: 10.1021/ja00053a020

2 Jia, M.; Seifert, A.; Berger, M.; Giegengack, H.; Schulze, S.; Thiel, W.R. Hybrid Mesoporous Materials with a Uniform Ligand Distribution: Synthesis, Characterization, and Application in Epoxidation Catalysis. *Chem. Mater.* **2004**, *16*, 877-882. DOI: 10.1021/cm031125g

3 Marler, B.; Oberhagemann, U.; Vortmann, S.; Gies, H. Influence of the sorbate type on the XRD peak intensities of loaded MCM-41. *Micropor. Mat.* **1996**, *6*, 375-383. DOI: 10.1016/0927-6513(96)00016-8

4 Soler-Illia, G.J.d.A.; Sanchez, C.; Lebeau, B.; Patarin, J. Chemical Strategies To Design Textured Materials: from Microporous and Mesoporous Oxides to Nanonetworks and Hierarchical Structures. *Chem. Rev.* **2002**, *102*, 4093-4138. DOI: 10.1021/cr0200062

[5] Zhou, Z.; Franz, A.W.; Bay, S.; Sarkar, B.; Seifert, A.; Yang, P.; Wagener, A.; Ernst, S.; Pagels, M.; Müller, T.J.J.; Thiel, W.R. Redox Active Mesoporous Hybrid Materials by In situ Syntheses with Urea-linked Triethoxysilylated Phenothiazines. *Chem. Asian J.* **2010**, *5*, 2001-2015. DOI: 10.1002/asia.201000098

[6] Tanev, P.T.; Pinnavaia, T.J. A Neutral Templating Route to Mesoporous Molecular Sieves. *Science* **1995**, *267*, 865-867. DOI: 10.1126/science.267.5199.865

[7] Sing, K.S.W.; Everett, D.H.; Haul, R.A.W.; Moscou, L.; Pierotti, R.A.; Rouquerol, J.; Siemieniewska, T. Reporting physisorption data for gas/solid systems with special reference to the determination of surface area and porosity. *Pure Appl. Chem.* **1985**, *57*, 603-619. DOI: 10.1351/pac198557040603

[8] Ravikovitch, P.I.; Odomhnaill, S.C.; Neimark, A.V.; Schüth, F.; Unger, K.K. Capillary Hysteresis in Nanopores: Theoretical and Experimental Studies of Nitrogen Adsorption on MCM-41. *Langmuir* **1995**, *11*, 4765-4772. DOI: 10.1021/la00012a030

[9] Rojas, F.; Kornhauser, I.; Felipe, C.; Esparza, J.M.; Cordero, S.; Dominguez, A.; Riccardo, J.L. *Phys. Chem. Chem. Phys.* **2002**, *4*, 2346-2355. DOI: 10.1039/B108785A

[10] Hunter, H.M.A.; Garcia-Bennett, A.E.; Shannon, I.J.; Zhou, W.; Wright, P.A. Capillary condensation in heterogeneous mesoporous networks consisting of variable connectivity and pore-size correlation. *J. Mater. Chem.* **2002**, *12*, 20-23. DOI: 10.1039/B108259K

[11] Zhao, X.S.; Lu, G.Q.; Whittaker, A.K.; Millar, G.J.; Zhu, H.Y. Comprehensive Study of Surface Chemistry of MCM-41 Using ^{29}Si CP/MAS NMR, FTIR, Pyridine-TPD, and TGA. *J. Phys. Chem. B* **1997**, *101*, 6525-6531. DOI: 10.1021/jp971366+

[12] Laughlin, R.B.; Joannopoulos, J.D. Phonons in amorphous silica. *Phys. Rev. B* **1977**, *16*, 2942. DOI: 10.1103/PhysRevB.16.2942

[13] Jia, M.J.; Seifert, A.; Thiel, W.R. Mesoporous MCM-41 Materials Modified with Oxodiperoxo Molybdenum Complexes: Efficient Catalysts for the Epoxidation of Cyclooctene. *Chem. Mater.* **2003**, *15*, 2174-2180. DOI: 10.1021/cm021380l
